# The m^6^A modification in cancer: roles, implications, and its potential in therapy

**DOI:** 10.1186/s43556-025-00314-2

**Published:** 2025-09-23

**Authors:** Yuyao Wei, Yang Wu, Chun Zhang, Mengling Yuan, Yuqi Sun, Mengran Li, Zili Zhang, Mei Guo

**Affiliations:** 1 School of Pharmacy, Nanjing University of Chinese Medicine, Nanjing, China; 2 School of Nursing, Nanjing University of Chinese Medicine, Nanjing, China; 3 Pancreas Center, Affiliated Hospital of Nanjing Medical University, Nanjing, China; 4 Affiliated Hospital of Nanjing University of Chinese Medicine, Nanjing, China

**Keywords:** M^6^A modification, Cancer diagnosis, Treatment strategies, Therapeutic target, Immunotherapy

## Abstract

N^6^-methyladenosine (m^6^A) serves as the dominant epitranscriptomic mark within eukaryotic mRNA transcripts, exerting pivotal regulatory functions in modulating mRNA structural integrity, translational efficiency, and splicing, thereby influencing gene expression patterns in cancer cells. m^6^A modification is recognized as a principal epigenetic determinant in driving malignant progression and fostering therapeutic resistance, making it crucial for advancing precision oncology. This review begins with a brief introduction to m^6^A modification, with a particular focus on its dynamic variability in distinct malignancies and clinical staging scenarios. Moreover, we underscore the critical functions of m^6^A methylation in cancer biology, including cancer-related molecular networks, cancer hallmarks, cancer stem cells, and the tumor microenvironment. We further outline the implications of m^6^A dysregulation in cancer, emphasizing the diagnostic potential of m^6^A regulators, the prognostic value of m^6^A, and the role of m^6^A in treatment resistance. Additionally, we analyze the potential of m^6^A modification in cancer therapy, encompassing the use of m^6^A inhibitors, combinations with existing cancer therapies, and personalized medicine approaches. Finally, we dissect the current limitations and future directions in m^6^A modification research, directing resources toward the development of high-throughput platforms for the dynamic monitoring of m^6^A modification in living systems. Overall, this review reinforces the central significance of m^6^A modification in cancer biology, emphasizing its transformative capacity to reshape cancer diagnostic paradigms and therapeutic intervention strategies.

## Introduction

RNA modifications are the chemical alterations discovered posttranscriptionally in RNA molecules and are typically mediated by specific enzymes. These modifications are essential for the structural and functional diversity of RNAs [1]. Currently, over 170 unique variants of RNA modifications have been identified, encompassing methylation, acetylation, and pseudouridylation [2]. These modifications are found in various RNA subclasses, including ribosomal RNA (rRNA), transfer RNA (tRNA), messenger RNA (mRNA), microRNA (miRNA), long noncoding RNA (lncRNA), and circular RNA (circRNA), where they exert pivotal influences on RNA structural integrity, alternative splicing processes, translational fidelity, and subcellular distribution dynamics [3]. Moreover, post-transcriptional RNA chemical alterations exert indispensable roles in sustaining cellular homeostasis and safeguarding transcriptional fidelity throughout the gene expression cascade [4]. Dysregulation of these modifications has been linked to diverse disease states, such as oncological disorders, neurodegenerative pathologies, and metabolic dysfunctions, underscoring their critical role in preserving cellular equilibrium [5, 6]. Therefore, studying RNA modifications provides profound understandings of the molecular mechanisms underlying cellular activity and disease pathogenesis and facilitates the identification of potential therapeutic targets for various diseases [7].

Since its discovery in the 1970 s, N^6^-methyladenosine (m^6^A) modification has been extensively studied for its biological functions, particularly in the regulation of gene expression [8]. The m^6^A methylation mechanism is orchestrated through a tripartite enzymatic system, i.e., methyltransferases, which catalyze the addition of m^6^A marks; demethylases, which reverse the methylation status via hydrolytic cleavage of the methyl group; and m^6^A-binding proteins, which decode these modifications to transduce downstream biological signals [9]. Emerging evidence underscores that m^6^A modification exerts a central regulatory role in tumorigenesis and malignant progression [10–13]. Dysregulated m^6^A modification is implicated in multiple malignancies, including glioblastoma [10], colorectal cancer [11], pancreatic cancer [12], and hepatocellular carcinoma [13]. During tumor initiation and progression, m^6^A modification regulates key oncogenes and tumor suppressors, consequently impacting cellular activities including proliferation, apoptosis, and differentiation [14]. Specifically, m^6^A modification increases the levels of oncogenes while suppressing the levels of tumor-suppressor genes, creating a protumorigenic environment [15]. Additionally, m^6^A methylation is intricately involved in the regulation of cancer stem cells, promoting their self-renewal, maintenance, and resistance to radiotherapy and chemotherapy, which further drives tumor progression [16, 17]. During the advanced phases of tumor progression, m^6^A modification influences cancer cell proliferation, migration, and invasion by modulating oncogenic signaling pathways within the tumor microenvironment (TME) [18, 19]. Despite the significance of m^6^A modification in cancer biology is achieving increasingly widespread recognition, the specific regulatory mechanisms underlying its dynamics remain poorly understood [18, 19]. Additional investigations are imperative to clarify the ways in which m^6^A-modifying enzymes and binding proteins contribute to tumor heterogeneity across different cancer types and individual patients. Moreover, while the prospect of leveraging m^6^A modifications for cancer treatment holds significant promise, rigorous clinical trials are essential to confirm the safety and therapeutic effectiveness of interventions centered on m^6^A methylation [18, 19]. Overcoming these hurdles is essential for fully harnessing the potential of m^6^A modification in cancer diagnosis and treatment.

This review focuses on the multifaceted role of m^6^A modification in cancer. First, we summarize the definition, chemical nature, and biological functions of m^6^A modification, with a particular focus on its dynamic variability in distinct malignancies and clinical staging scenarios. Moreover, we discuss how m^6^A modification contributes to tumor development and metastasis and further highlight the regulatory mechanisms of m^6^A modification in cancer. Additionally, we evaluate the therapeutic potential of m^6^A modification in cancer, emphasizing the feasibility of targeting m^6^A-related enzymes and the importance of developing m^6^A inhibitors. Finally, we integrate m^6^A modification with other cancer therapies and propose future directions for its application in personalized medicine, emphasizing its potential as a novel therapeutic strategy.

## Understanding m^6^A modification

### Definition and chemical nature of m^6^A modification

**Fig. 1:**
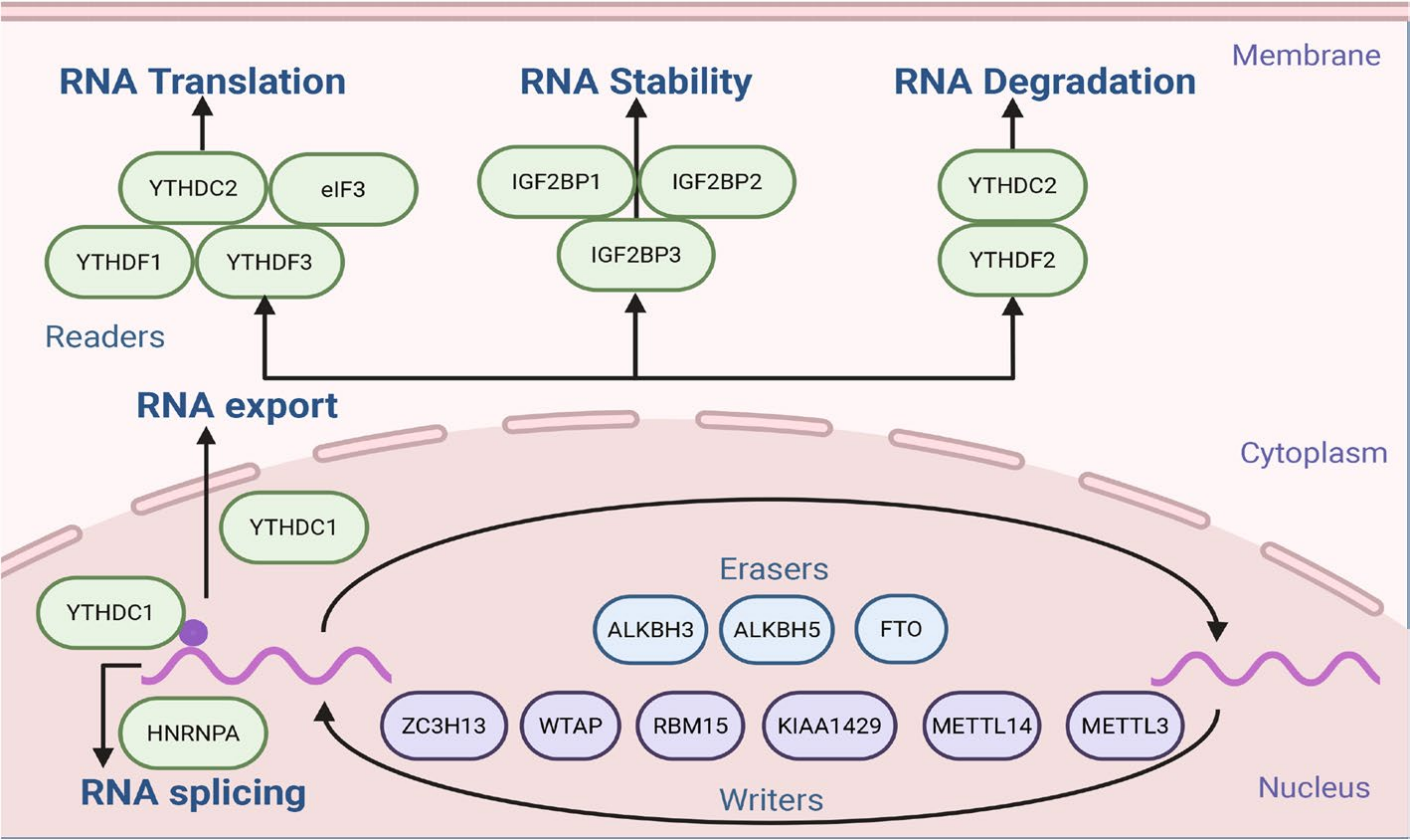
Understanding m^6^A modification in cancer. The m^6^A modification is dynamically regulated by three key classes of enzymes, such as writers, erasers, and readers. Writers include ZC3H13, WTAP, RBM15, KIAA1429, METTL14, and METTL3, which can catalyze m^6^A methylation. Erasers include ALKBH3, ALKBH5 and FTO, which can remove m^6^A modification. Readers include YTHDF1/2/3, YTHDC1/2, IGF2BP1/2/3, eIF3, and HNRNPA, which can recognize m^6^A sites and induce RNA splicing, export, translation, stability, and degradation

m^6^A modification is defined as methylation at the sixth nitrogen atom of adenosine within RNA transcripts [20]. It constitutes a broadly distributed and flexible posttranscriptional RNA modification that occurs mainly near conserved motifs (such as RRACH, R = A/G, and H = A/C/U) and is usually focused around the mRNA termination codon [20]. Studies have shown that m^6^A in coding regions (CDSs) works faster and more efficiently than that in 3' untranslated regions (3'-UTRs) [21, 22]. m^6^A modification affects nearly all phases of mRNA processing, from maturation in the nuclear compartment to protein synthesis and turnover in the cytosol, which is essential for the precise control of transcriptional output [23, 24]. Within unpaired RNA segments, the presence of m^6^A induces heightened π-orbital stacking interactions compared with those of unmodified residues, resulting in enthalpic stabilization of the surrounding tertiary structure via reduced conformational flexibility [25]. m^6^A modification promotes the translation of mRNAs by engaging eukaryotic initiation factors (eIFs), thereby markedly elevating the rate of capped mRNA translation [26]. In addition, m^6^A modification affects various physiological functions, such as the immune response and inflammatory response [27]. m^6^A methylation is reversible and dynamic, and this process is regulated by three key types of enzymes: writers (methyltransferases), erasers (demethylases), and readers (m^6^A-binding proteins). Writers are responsible for catalyzing the formation of m^6^A, erasers can remove m^6^A, and readers can specifically bind to m^6^A sites and mediate downstream effects on RNA function (Fig. 1). The m^6^A epitranscriptomic control system enables the context-specific modulation of RNA metabolic functions through nucleotide-specific combinatorial logic [28]. This adaptive interface with cellular microenvironments allows m^6^A methylation to serve as a molecular rheostat, integrating extracellular stimuli with intracellular regulatory networks to calibrate gene expression outputs with remarkable spatiotemporal precision [28]. m^6^A modification can rapidly and reversibly regulate RNA function. Unlike gene mutations, m^6^A modification does not change the basic sequence of RNA but rather controls its processing and final fate [29]. This property makes it an efficient and flexible regulatory mechanism that is capable of responding quickly to changes in cellular conditions or environmental stimuli. Importantly, this reversible modification is not limited to individual RNA molecules but can affect the entire transcriptome, making it a powerful tool for fine-tuning gene expression in various biological processes [29].

### Key enzymes involved in m^6^A modification

To date, over 20 unique m^6^A-associated enzymes have been characterized [30]. These molecular players clearly exhibit functional specialization. These catalytic and recognition modules synergistically regulate the m^6^A epigenomic code, thereby orchestrating critical posttranscriptional regulatory cascades that determine RNA fate through modulated splicing kinetics, nuclear‒cytoplasmic partitioning, turnover rates, structural integrity, and ribosome engagement (Table 1).

**Table 1:** Type and function of m^6^A enzymes

Type	m^6^A enzyme	Function	Reference
Writers	METTL3	The core enzyme that exerts the catalytic activity of the methyltransferase	[31]
	METTL14	The structural support part that maintains the stability of the methyltransferase	[31]
	WTAP	The bridging protein that promotes the methyltransferase recruitment to mRNA targets	[32]
	KIAA1429	It recruits the methyltransferase to guide regions for selective m^6^A modification	[33]
	ZC3H13	It anchors the methyltransferase to achieve RNA m^6^A methylation in the nucleus	[34]
Erasers	FTO	It exerts the catalytic activity of the demethylase	[35, 36]
	ALKBH5	It exerts the catalytic activity of the demethylase	[37–39]
	ALKBH3	It exerts the catalytic activity of the demethylase	[40–42]
Readers	YTHDF1	It facilitates the stability of m^6^A-modified RNA	[43]
	YTHDF2	It facilitates the degradation of m^6^A-modified RNA	[44]
	YTHDF3	It facilitates the translation and decay of m^6^A-modified RNA	[45]
	YTHDC1	It promotes the splicing and nuclear export of m^6^A-modified RNA	[46, 47]
	YTHDC2	It promotes the transcription efficiency of m^6^A-modified RNA	[48, 49]
	IGF2BP1	It enhances the stability and translation efficiency of m^6^A-modified RNA	[50]
	IGF2BP2	It enhances the stability and translation efficiency of m^6^A-modified RNA	[50]
	eIF3	It mediates cap-independent translation of m^6^A-modified RNA	[51]
	HNRNPA2B1	It mediates the splicing of m^6^A-modified RNA	[52]

#### Writers

Writers represent a class of sophisticated catalytic enzymes capable of installing m^6^A on RNA nucleotides through a highly coordinated series of biochemical reactions. Central to this process is the methyltransferase complex (MTC), which includes methyltransferase-like 3 (METTL3), METTL4, METTL14, Wilms’ tumor 1-associated protein (WTAP), vir-like m^6^A methyltransferase-associated protein (KIAA1429), and zinc finger CCCH-type containing 13 (ZC3H13) [31]. These components do not function in isolation but rather engage in dynamic protein‒protein interactions to form a functional holoenzyme. Among them, METTL3 is the core enzyme that performs the catalytic activity. METTL14 is a structural support protein that can maintain the stability of the MTC and promote the binding of RNA substrates. Its interaction with METTL3 exerts a synergistic enhancement on MTC's catalytic capacity [31]. WTAP, an adaptor protein, engages in interactions with METTL3 and METTL14, potentially modulating the targeting of the MTC to mRNA substrates [32]. KIAA1429 mobilizes the enzymatic core subunits METTL3/METTL14/WTAP to direct targets for site-specific m^6^A methylation, establishing it as the most substantial protein identified within the complex [33]. ZC3H13 interacts with WTAP, and its main function is to anchor the ZC3H13–WTAP–Virilizer–Hakai assembly within the nuclear compartment to mediate RNA m^6^A modification of RNA transcripts [34].

#### Erasers

Erasers function as critical modulators by catalyzing the excision of methyl moieties from m^6^A-modified residues. This enzyme family comprises three primary members, including ALKB homolog 3 (ALKBH3), ALKBH5, and fat mass and obesity-associated protein (FTO). The first discovered m^6^A demethylase is FTO in eukaryotic cells, and elevated FTO expression is mechanistically linked to reduced m^6^A abundance through its oxidative demethylation activity [35]. Notably, oncogenic reprogramming pathways frequently hijack this enzymatic activity, with aberrant FTO overexpression serving as a recurrent biomarker across multiple malignancy subtypes. Functional studies have elucidated the dual oncogenic capacities of FTO, demonstrating its ability to both promote neoplastic proliferation through destabilization of tumor suppressor transcripts and potentiate metastatic dissemination via the epigenetic reprogramming of invasion-associated gene networks [36]. Emerging evidence implicates ALKBH5-catalyzed m^6^A demethylation as a pivotal modulator of transcriptional output, operating through the multimodal modulation of posttranscriptional RNA processing events, including splicing, stability, and translational efficiency [37]. This regulatory axis is important in oncogenic contexts, where dysregulated ALKBH5 expression exerts context-dependent tumor-promoting or tumor-suppressing effects [37]. Remarkably, the functional output and clinical relevance of ALKBH5 exhibit remarkable heterogeneity across distinct cancer subtypes, underscoring the complexity of m^6^A-mediated epigenetic control in malignancy [38]. Initial characterization revealed that ALKBH3 is a demethylase for N^1^-methyladenosine and 3-methylcytosine and plays roles in oncogenic processes, including proliferation, migration, and invasion [39]. More recent mechanistic insights have revealed an expanded catalytic repertoire, as ALKBH3 has also been shown to mediate the demethylation of m^6^A modification sites [40]. This newly elucidated function increases the ribosome occupancy and translational efficiency of target mRNAs, thereby establishing a direct link between ALKBH3 and tumor growth promotion [40].

#### Readers

Readers mediate diverse phases of RNA processing, including YTH domain family (YTHDC1/2, YTHDF1/2/3), insulin-like growth factor 2 mRNA-binding protein family (IGF2BP1/2/3), heterogeneous nuclear ribonucleoprotein A2/B1 (HNRNPA2B1), and eIF3 [41, 42]. Upon engagement with these modified nucleotides, the protein undergoes conformational changes that facilitate the recruitment and activation of downstream effector molecules [41, 42]. This coordinated interplay between m^6^A recognition and effector molecule engagement is critical for the precise modulation of diverse biological processes, underscoring the protein functions as a central coordinator in posttranscriptional regulatory networks [41, 42]. Among them, YTHDF1 detects m^6^A-modified mRNA transcripts, enhancing their translation efficiency and thereby elevating protein output [43]. YTHDF2 functions as a principal mediator in mRNA decay, accelerating transcript turnover and reducing mRNA stability [44]. YTHDF3 collaborates with YTHDF1 to boost mRNA translation while modulating its functional activity [45]. YTHDC1 orchestrates mRNA splicing by engaging spliceosomal components and facilitating the association of transcripts with target genomic loci [46]. In addition, YTHDC1 mediates the nuclear export of m^6^A-methylated mRNAs [47]. YTHDC2 enhances the transcriptional productivity of mRNA through its ability to identify m^6^A methylation within the coding sequence. The depletion of YTHDC2 results in decreased protein synthesis [48]. YTHDC2 can also function as a suppressor of transcriptional output at the transcriptional phase through the acceleration of mRNA decay [49]. IGF2BPs function as mRNA stabilizers by enhancing post-transcriptional integrity and suppressing transcript degradation [50]. eIF3 can selectively bind to m^6^A-containing RNA and facilitate cap-independent translation initiation of mRNA [51]. HNRNPA2B1 engages specific subsets of nuclear pre-mRNA transcripts and modulates the differential exon utilization within discrete cohorts of transcripts through alternative splicing pathways [52].

### Biological functions of m^6^A modification

Recent studies underscore the pivotal significance of m^6^A modification in multiple tumor types, driven by the bifunctional regulatory mechanisms of writers and erasers through dynamic methylation-demethylation equilibrium [53, 54]. The complex METTL3/METTL14 operates as the principal methyltransferase in m^6^A modification, catalyzing the installation of m^6^A marks on mRNAs encoding oncogenes or tumor suppressor genes. These epigenetic marks serve as dynamic molecular switches that are specifically recognized by reader proteins, triggering downstream molecular cascades [55]. When oncogene mRNAs are targeted, erasers' activity eliminates m^6^A signals to prevent aberrant reader recruitment and subsequent oncogene activation [56]. Paradoxically, the same erasers can act on tumor suppressor transcripts by removing protective m^6^A marks, thereby disrupting normal mRNA turnover mechanisms and causing the aberrant accumulation of tumor suppressor proteins [56]. This intricate interplay between methylation and demethylation underscores the context-dependent oncogenic potential of m^6^A dysregulation, where imbalances at any node of this regulatory axis may contribute to malignant transformation and tumor progression [57]. Emerging evidence reveals the central significance of m^6^A modification as a indispensable epigenetic regulator of gene expression, cellular development, and cancer pathogenesis [57]. As research progresses, the molecular mechanisms and biological ramifications of m^6^A modulation are being progressively elucidated, revealing novel therapeutic targets and diagnostic paradigms for cancer intervention.

## Role of m^6^A modification in cancer biology

**Fig. 2:**
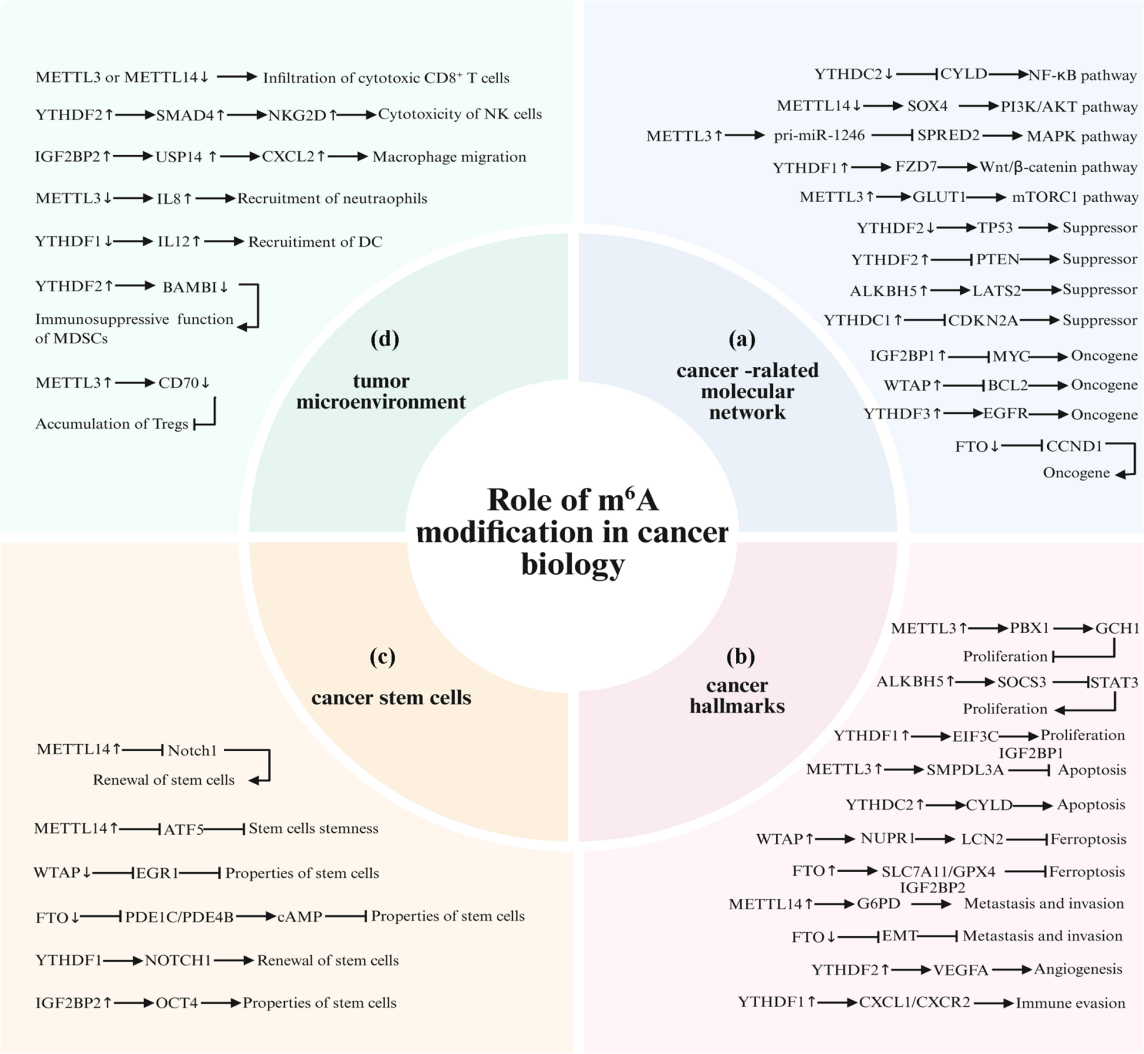
Role of m^6^A modification in cancer biology. The role of m^6^A modification in cancer biology includes the cancer-related molecular networks, cancer hallmarks, cancer stem cells, and the tumor microenvironment. **a** m^6^A modification and cancer-related molecular networks. **b** m^6^A modification in cancer hallmarks. **c** m^6^A modification and cancer stem cells. **d** m^6^A modification and the tumor microenvironment

m^6^A modification profoundly influences cancer biology through diverse mechanisms, including cancer-related signaling reprogramming, cancer hallmark acquisition, cancer stem cell maintenance, and TME remodeling (Fig. 2). Comprehensive dissection of these context-dependent m^6^A-driven mechanisms holds transformative potential for developing precision epigenomic therapies targeting the RNA modification landscape in human malignancies (Table 2).

**Table 2:** Roles and key mechanisms of m^6^A regulators in cancer

Type	m^6^A regulator	Expression	Function	Mechanism	Cancer type	Reference
Writer	METTL3	Upregulation	Oncogene	It increases pri-miR221/222 maturation	Bladder cancer	[58]
Writer	METTL3	Upregulation	Oncogene	It increases BHLHE41/CXCL1/CXCR2 axis	Colorectal cancer	[59]
Writer	METTL3	Downregulation	Tumor suppressor	It decreases neutrophil infiltration	Papillary thyroid cancer	[60]
Writer	METTL3	Upregulation	Oncogene	It increases pentose phosphate pathway	Hepatocellular Carcinoma	[61]
Writer	METTL14	Downregulation	Tumor suppressor	It increases SOX4 m^6^A modification	Colorectal cancer	[62]
Writer	METTL14	Downregulation	Tumor suppressor	It increases Notch1 m^6^A modification	Bladder cancer	[63]
Writer	METTL14	Downregulation	Tumor suppressor	It increases USP48 mRNA stability	Hepatocellular carcinoma	[64]
Writer	WTAP	Upregulation	Oncogene	It increases IL6/STAT3 pathway	Gastric cancer	[65]
Writer	WTAP	Upregulation	Oncogene	It increases NRF2 m^6^A modification	Bladder cancer	[66]
Writer	WTAP	Upregulation	Oncogene	It increases ENO1 m^6^A modification	Breast cancer	[67]
Writer	WTAP	Upregulation	Oncogene	It increases MAPK signaling	Colorectal cancer	[68]
Writer	WTAP	Upregulation	Oncogene	It increases HuR/ETS1/P21/P27 axis	Hepatocellular carcinoma	[69]
Writer	KIAA1429	Upregulation	Oncogene	It decreases WEE1 m^6^A modification	Colorectal cancer	[70]
Writer	KIAA1429	Upregulation	Oncogene	It increases GATA3 m^6^A modification	Liver cancer	[71]
Writer	KIAA1429	Upregulation	Oncogene	It increases ENO1 expression	Ovarian cancer	[72]
Writer	KIAA1429	Upregulation	Oncogene	It decreases RASD1 mRNA stability	Gastric cancer	[73]
Writer	KIAA1429	Upregulation	Oncogene	It increases JNK/MAPK pathway	Lung cancer	[74]
Eraser	FTO	Downregulation	Tumor suppressor	It decreases APOE mRNA stability	Papillary thyroid cancer	[75]
Eraser	FTO	Upregulation	Oncogene	It increases SLC7A5 stability	Gastric cancer	[76]
Eraser	FTO	Upregulation	Oncogene	It decreases ferroptosis pathway	Colorectal cancer	[77]
Eraser	FTO	Upregulation	Oncogene	It increases GPNMB mRNA stability	Hepatocellular carcinoma	[78]
Eraser	FTO	Upregulation	Oncogene	It increases NEDD4 mRNA stability	Pancreatic cancer	[79]
Eraser	ALKBH5	Downregulation	Tumor suppressor	It inhibits cancer by activating PER1	Pancreatic cancer	[80]
Eraser	ALKBH5	Upregulation	Oncogene	It decreases ITGB1 m^6^A modification	Ovarian cancer	[81]
Eraser	ALKBH5	Downregulation	Tumor suppressor	It decreases CD276 mRNA stability	Colorectal cancer	[82]
Eraser	ALKBH5	Upregulation	Oncogene	It decreases TACC3 m^6^A modification	Acute myeloid leukemia	[83]
Eraser	ALKBH5	Downregulation	Tumor suppressor	It decreases GLUT4 m^6^A modification	Breast cancer	[84]
Reader	YTHDF1	Upregulation	Oncogene	It increases PLK1 mRNA stability	Prostate cancer	[85]
Reader	YTHDF1	Upregulation	Oncogene	It increases FZD7 translation	Gastric cancer	[86]
Reader	YTHDF1	Upregulation	Oncogene	It increases EIF3C translation	Ovarian cancer	[87]
Reader	YTHDF1	Upregulation	Oncogene	It increases ARHGEF2 mRNA stability	Colorectal cancer	[88]
Reader	YTHDF1	Upregulation	Oncogene	It increases EZH2 and CDH11 translation	Breast cancer	[89]
Reader	YTHDF2	Upregulation	Oncogene	It decreases GSK3β mRNA stability	Colorectal cancer	[90]
Reader	YTHDF2	Upregulation	Oncogene	It decreases SETD7 and KLF4 mRNA	Bladder cancer	[91]
Reader	YTHDF2	Upregulation	Oncogene	It decreases AC026691.1 mRNA	Gastric cancer	[92]
Reader	YTHDF2	Upregulation	Oncogene	It increases OCT4 expression	Liver cancer	[93]
Reader	YTHDF3	Upregulation	Oncogene	It increases Notch2 translation	Breast cancer	[94]
Reader	YTHDF3	Downregulation	Tumor suppressor	It decreases P4HA2 mRNA stability	Papillary thyroid cancer	[95]
Reader	YTHDF3	Upregulation	Oncogene	It decreases ZFP41 mRNA stability	Hepatocellular carcinoma	[96]
Reader	YTHDC1	Downregulation	Tumor suppressor	It decreases TSFM mRNA stability	Ovarian cancer	[97]
Reader	YTHDC1	Upregulation	Oncogene	It decreases UBE3A expression	Colorectal cancer	[98]
Reader	YTHDC1	Downregulation	Tumor suppressor	It decreases FSP1 mRNA stability	Lung cancer	[99]
Reader	YTHDC2	Downregulation	Tumor suppressor	It decreases hedgehog pathway	Endometrial cancer	[100]
Reader	YTHDC2	Downregulation	Tumor suppressor	It decreases Akt pathway	Papillary thyroid cancer	[101]
Reader	YTHDC2	Downregulation	Tumor suppressor	It increases ID3 expresssiom	Non-small cell lung cancer	[102]
Reader	YTHDC2	Upregulation	Oncogene	It increases YAP mRNA translation	Gastric cancer	[103]
Reader	YTHDC2	Downregulation	Tumor suppressor	It decreases LIMK1 mRNA stability	Colorectal cancer	[104]
Reader	IGF2BP1	Downregulation	Tumor suppressor	It decreases MYC mRNA stability	Gastric cancer	[105]
Reader	IGF2BP1	Upregulation	Oncogene	It increases CDC25A mRNA stability	Cervical cancer	[106]
Reader	IGF2BP1	Upregulation	Oncogene	It increases PARK7 mRNA stability	Hepatocellular carcinoma	[107]
Reader	IGF2BP1	Upregulation	Oncogene	It increases INHBA mRNA stability	Esophageal squamous cancer	[108]
Reader	IGF2BP1	Upregulation	Oncogene	It increases BUB1B mRNA stability	Non-small cell lung cancer	[109]
Reader	IGF2BP2	Upregulation	Oncogene	It increases CDKN2A mRNA stability	Cutaneous T-cell lymphomas	[110]
Reader	IGF2BP2	Upregulation	Oncogene	It increases HMGA1 mRNA stability	Colorectal cancer	[111]
Reader	IGF2BP2	Upregulation	Oncogene	It increases CDK6 translation	Triple-negative breast cancer	[112]
Reader	IGF2BP2	Upregulation	Oncogene	It increases HMMR mRNA stability	Non-small cell lung cancer	[113]
Reader	IGF2BP2	Upregulation	Oncogene	It increases CDH12 mRNA stability	Thyroid carcinoma	[114]
Reader	IGF2BP2	Upregulation	Oncogene	It increases OCT4 mRNA stability	Esophageal squamous cell carcinoma	[93]
Reader	IGF2BP2	Upregulation	Oncogene	It increases glutamine metabolism pathways	Myeloid leukemia	[115]
Reader	IGF2BP2	Upregulation	Oncogene	It increases SLC1A5/mTORC1 axis	Pancreatic cancer	[116]
Reader	HNRNPA2B1	Upregulation	Oncogene	It increases circCDYL/EIF4A3/PHF8 axis	Colorectal cancer	[117]
Reader	HNRNPA2B1	Upregulation	Oncogene	It increases miR-21-5p/PTEN axis	Non-small cell lung cancer	[118]
Reader	HNRNPA2B1	Upregulation	Oncogene	It increases ALYREF/NXF1 complex	Breast cancer	[119]

### m^6^A modification and cancer-related molecular networks

#### Regulation of key pathways

Recent scientific findings illuminate the multifaceted regulatory complexity of m^6^A modification in cellular signaling pathways, rendering this epitranscriptomic mark a pivotal target for deciphering pathogenic molecular mechanisms and developing targeted therapeutic interventions. Importantly, typical signaling pathways regulated by m^6^A modification include the nuclear factor-κB (NF-κB) pathway, phosphoinositide 3 kinase/protein kinase B (PI3K/AKT) pathway, mitogen-activated protein kinase (MAPK) pathway, wingless-related integration site (Wnt) pathway, and mechanistic target of rapamycin (mTOR) pathway [120–122]. These signaling pathways exert pivotal regulatory functions in cellular growth, proliferative signaling, lineage commitment, and programmed cell death, with their dysregulated regulation mediated by m^6^A modification being strongly implicated in the pathogenesis of multiple disorders, particularly oncogenic transformation. Specifically, the activation of the tumor necrosis factor receptor-associated factor 6 (TRAF6)/NF-κB/fibroblast growth factor 18 (FGF18) pathway is required for gastric cancer cell proliferation through METTL3-mediated m^6^A methylation [120]. METTL3 promotes bladder cancer progression via the AF4/FMR2 family member 4 (AFF4)/NF-κB signaling network. Two key regulators of the NF-κB pathway, namely IKBKB and RELA, have been identified as direct targets of METTL3-catalyzed m^6^A methylation [121]. METTL14 suppression attenuates glutaminase 2 (GLS2) expression by impairing YTHDF1-mediated translational efficiency in an m^6^A-dependent manner, thereby fostering an oxidative stress microenvironment and recruiting Cx3cr1⁺ Ccr2⁺ monocyte-derived macrophages. Concurrently, METTL14 deficiency activates S100A4⁺ Macrophages through the myeloid differentiation primary response 88 (MyD88)/NF-κB signaling, ultimately driving hepatocellular carcinoma progression [122]. The cylindromatosis (CYLD)/NF-κB pathway has been confirmed to be downstream of YTHDC2, and this axis is mediated by m^6^A modification in lung cancer. YTHDC2 exerts its anti-tumor effects via the CYLD/NF-κB signaling pathway, a process modulated by m^6^A modification [123].

The PI3K/AKT pathway acts as a pivotal intracellular signaling network, exhibiting frequent deregulation across multiple human malignancies, and serving as a pivotal axis in driving tumor progression, therapeutic resistance, and metastatic potential. METTL14 may inhibit the malignant process of colorectal cancer via PI3K/AKT pathway and epithelial‒mesenchymal transition (EMT) induced by SRY-related high-mobility-group box 4 (SOX4) [62]. YTHDF1 facilitates the efficient translation of polo-like kinase 1 (PLK1) through m^6^A-methylated PLK1 mRNA levels, thereby augmenting hyperactivation of the PI3K/AKT signaling cascade. This YTHDF1–PLK1–PI3K–AKT signaling axis plays a pivotal role in prostate cancer progression and represents a promising therapeutic target for clinical intervention [85]. Similarly, METTL3 catalyzes m^6^A modification on pri-miR-1246, thereby enhancing its maturation process. The sprouty-related EVH1 domain-containing protein 2 (SPRED2) is identified as a downstream effector of miR-1246, and its downregulation abrogates the suppression of MAPK pathway activity. METTL3 overexpression facilitates colorectal cancer cell metastatic progression through the miR-1246–SPRED2–MAPK signaling axis [124]. Furthermore, WTAP interacts with carbonic anhydrase IV (CA4) to induce WTAP protein degradation through polyubiquitination, which may inhibit colon cancer development by suppressing the Wnt signaling pathway [125]. Moreover, YTHDF1 promotes the translation of the key Wnt receptor Frizzled7 (FZD7) through m^6^A recognition, while a gain-of-function mutation in YTHDF1 elevates FZD7 expression by stabilizing its mRNA, leading to constitutive activation of the Wnt/β-catenin signaling cascade and driving gastric tumorigenesis [86]. YTHDF2 enhances AXIN1 mRNA degradation through m^6^A-dependent recognition, thereby reducing AXIN1 protein levels and activating the Wnt/β-catenin signaling cascade. This YTHDF2–AXIN1–Wnt axis drives lung adenocarcinoma progression by augmenting tumor cell proliferative capacity and metastatic potential [126]. miRNA-6125 interacts with the 3'-UTR of YTHDF2 mRNA, inhibiting YTHDF2 protein synthesis and consequently stabilizing m^6^A-modified glycogen synthase kinase 3β (GSK3β) transcripts. Elevated GSK3β suppresses Wnt/β-catenin/cyclin D1 signaling through downregulation of pathway effectors, triggering G0/G1 cell cycle arrest and suppressing colorectal cancer cell proliferation [90].

The mTOR pathway operates as a central regulatory hub for cancer cell growth, metabolic reprogramming, and survival. METTL3 mediates m^6^A-dependent translational regulation of glucose transporter 1 (GLUT1), thereby enhancing glucose uptake and lactate production. This metabolic reprogramming consequently drives activation of the mTORC1 signaling cascade, ultimately contributing to colorectal carcinogenesis [127]. ALKBH5-mediated elevation of DNA-damage-inducible transcript 4 antisense RNA 1 (DDIT4-AS1) sustains pancreatic cancer stem-like properties and reduces chemotherapeutic responsiveness through mTOR pathway activation. Mechanistically, DDIT4-AS1 prevents the interaction between the nonsense-mediated mRNA decay factor (SMG5) and RNA helicase/ATPase (UPF1), thereby promoting UPF1 phosphorylation. This molecular event decreases DDIT4 mRNA stability, leading to constitutive activation of the mTOR signaling cascade [128]. Beyond canonical signaling cascades, m^6^A modifications modulate other signaling pathways. METTL3 enhances the six transmembrane epithelial antigen of prostate 2 (STEAP2) mRNA stability through m^6^A-dependent post-transcriptional regulation, thereby elevating STEAP2 expression levels. The METTL3–STEAP2 axis functions as a tumor suppressor in papillary thyroid carcinoma by inhibiting EMT program and suppressing hedgehog signaling through transcriptional repression of its effectors [129]. Moreover, the lncRNA AGAP2-AS1 interacts with WTAP to facilitate assembly of the WTAP/METTL3/METTL14 complex. Concurrently, AGAP2-AS1 enhances STAT3 mRNA stability and triggers interleukin-6 (IL-6)/STAT3 pathway activation. Dysregulation of the AGAP2–AS1–WTAP–STAT3 axis drives gastric carcinogenesis by enhancing tumor cell proliferative capacity and metastatic potential [65]. FTO catalyzes m^6^A demethylation of Apolipoprotein E (APOE) mRNA, which is subsequently bound and stabilized by IGF2BP2, thereby suppressing glycolysis in papillary thyroid cancer through modulation of the IL-6/STAT3 signaling cascade [75]. In addition, ALKBH5 posttranscriptionally activates period circadian regulator 1 (PER1) in an m^6^A-YTHDF2-dependent mechanism, with subsequent PER1 upregulation driving reactivation of the ATM–CHK2–CDC25C signaling cascade to suppress pancreatic cancer cell proliferation [80]. Overall, the regulation of key pathways by m^6^A methylation exhibits an intricate association with the physiological and pathological states of cancer cells. The dynamic interplay among three key types of m^6^A enzymes results in the construction of a complex regulatory web that fine-tunes the activity of signaling pathways.

#### Impact on tumor suppressor genes and oncogenes

Epigenetic m^6^A methylation exerts a critical function in the adaptive modulation of tumor-suppressive genes and oncogenes, resulting in a dual regulatory effect that profoundly influences tumorigenesis. Notably, m^6^A modification can either facilitate the degradation of tumor suppressors or impede their translation, ultimately diminishing their tumor-suppressive functions. Conversely, it may stabilize the mRNA transcripts of oncogenes or increase their translational efficiency, thereby fostering tumor initiation and progression [130]. This dynamic regulation underscores the complexity of m^6^A methylation in cancer biology, thus positioning it as a pivotal domain of scientific investigation toward cancer pathogenesis and developing targeted therapies. Importantly, the typical tumor suppressors regulated by m^6^A modification include tumor protein 53 (TP53), phosphatase and tensin homolog (PTEN), large tumor suppressor homolog 2 (LATS2) and cyclin-dependent kinase inhibitor 2 A (CDKN2A) [130]. Specifically, two contiguous m^6^A modification sites reside within CDS of TP53, separated by a mere two-nucleotide interval. These sites are potentially recognized by YTHDF2 in a concomitant or competitive manner. m^6^A methylation likely modulates TP53 mRNA stability, thereby determining the malignant phenotypic traits of colorectal cancer [131]. Moreover, YTHDF2 interacts with clustered m^6^A-modified regions on PTEN mRNA, eliciting phase separation and forming membraneless condensates that inhibit translation initiation, thereby diminishing PTEN protein expression. This poly-m^6^A-mediated mechanism drives arsenite-induced malignant transformation through sustained repression of PTEN [132]. METTL14 expression is attenuated in renal cell carcinoma tissues, and ectopic overexpression of METTL14 inhibits cell proliferative capacity and invasive migration potential. METTL14 overexpression elevates PTEN transcript modification density, subsequently enhancing its stability and upregulating protein expression levels [133]. Additionally, m^6^A modification of LATS2 can enhances its recognition by YTHDF2 and subsequent decay. Attenuated LATS2 abundance accelerates hepatoblastoma tumorigenesis through suppression of ferroptotic execution [134]. Phosphorylated ALKBH5 specifically removes methyl groups from LATS2 mRNA, thereby enhancing its stability through inhibition of YTHDF2-mediated decay. Notably, LATS2 knockdown inhibits self-renewal capacity of glioblastoma stem cell (GSC) in a yes-associated protein (YAP)-independent manner, suggesting a divergent regulatory axis in stemness maintenance [135]. Furthermore, circMET is delivered to the cytosol by YTHDC1. circMET functions as a molecular scaffold that physically interacts with YTHDF2, facilitating its recruitment to CDKN2A mRNA and thereby promoting m^6^A-dependent decay of this tumor suppressor transcript [136]. Hypomethylated CDKN2A transcripts fail to recruit IGF2BP2, leading to YTHDF2-mediated decay of this tumor suppressor mRNA. The resultant downregulation of CDKN2A drives neoplastic progression in cutaneous T-cell lymphoma [110].

In contrast, prototypical oncogenes influenced by m^6^A-mediated mechanisms include myelocytomatosis oncogene (MYC), B-cell lymphoma 2 (BCL2), epidermal growth factor receptor (EGFR) and cyclin D 1 (CCND1) [130]. Specifically, METTL3-catalyzed m^6^A methylation enhances MYC mRNA translational efficiency, thereby driving cervical carcinoma tumorigenesis through MYC-dependent cell cycle dysregulation [137]. miR-96 suppresses AMP-activated protein kinase alpha 2 (AMPKα2), inducing FTO upregulation and subsequent MYC expression enhancement via prevention of its m^6^A modification, thereby mediating pro-proliferative and anti-apoptotic effects in colorectal cancer [138]. IGF2BP1 exerts tumor-suppressive effects in gastric cancer development by suppressing MYC expression through an m^6^A-mediated mechanism [105]. Interestingly, neuropilin-1 (NRP1), a transmembrane glycoprotein overexpressed in breast cancer cells, attenuates ionizing radiation (IR)-induced apoptotic response by suppressing BCL2 transcription through WTAP-mediated m^6^A methylation [139]. In addition, YTHDF3 increases the stability and translation of m^6^A-methylated EGFR transcripts and drives hepatocellular carcinoma advancement [140]. Importantly, FTO knockdown markedly attenuates CCND1 expression via YTHDF2-mediated recognition and subsequent mRNA decay, thereby arresting cell cycle progression [141]. Notably, m^6^A regulators not only act as critical modulators in the dynamic modulation of tumor suppressors and oncogenes but also exhibit dual roles as tumor suppressors or oncogenes in cancer pathogenesis. YTHDC2 functions as a tumor suppressor in endometrial carcinoma, with its silencing promoting endometrial cancer cell proliferation [100]. KIAA1429 exhibits oncogenic activity in gastric cancer by stabilizing c-Jun mRNA via an m^6^A-independent pathway, and its overexpression promotes gastric cancer cell proliferation [142]. In addition, KIAA1429 functions as An oncogenic driver in colorectal carcinoma by elevating Sirtuin 1 expression [143]. Overall, m^6^A modification regulates tumor suppressors and oncogenes via regulating pre-mRNA splicing, mRNA stability, and protein synthesis. Future research should focus on targeting m^6^A regulatory enzymes to enhance therapeutic responses, leveraging tools such as MeRIP-seq for precision mapping.

### m^6^A modification in cancer hallmarks

The hallmarks of cancer represent a framework for understanding the multifaceted biological capabilities acquired by malignant cells during tumorigenesis. This review delves into serval pivotal hallmarks, namely, infinite cell proliferation, resistance to cell death, invasion and metastasis, angiogenesis, and immune evasion, and highlights their molecular mechanisms and therapeutic implications [144].

#### Cell proliferation

A defining hallmark of cancer cells is their acquisition of sustained proliferative signaling, driven by compromised regulatory checkpoints and dysregulated growth-promoting pathways. Contemporary research underscores that the modulatory function of m^6^A methylation in regulating cancer cell proliferation dynamics has drawn substantial academic focus, positioning it as a pivotal investigative frontier in oncology [145]. m^6^A methyltransferases exert different effects on the dynamics of cancer cell proliferative behavior by regulating a spectrum of target genes. Specifically, METTL3-induced m^6^A methylation of Matrix metallopeptidase 9 (MMP9) mRNA can promote colorectal cancer proliferation [146]. METTL3 catalyzes the m^6^A modification of lncRNA SNHG1, enhancing its RNA stability and subsequently driving tumorigenic proliferation of colorectal cancer cells [147]. Notably, METTL3 functions as An oncogenic driver in bladder cancer by engaging in a functional interaction with DiGeorge syndrome critical region gene 8 (DGCR8), thereby enhancing the biogenesis of miR-221/222 through positive regulation of pri-miR221/222 processing [58]. The lncRNA 00460/IGF2BP2 complex May promote colorectal cancer cell proliferation by mediating high mobility group AT-hook 1 (HMGA1) transcript stability through METTL3-dependent m^6^A modification [111]. Moreover, the METTL3–YTHDF1–m^6^A signaling axis functions as a pivotal driver in ovarian cancer progression. This axis engages in a functional interaction with discoidin domain receptor 2 (DDR2) mRNA, enhancing the expression of the pro-tumorigenic protein DDR2 and thereby fueling ovarian cancer malignancy [148]. The METTL3–YTHDF2–m^6^A signaling axis orchestrates the turnover of transcripts encoding the kruppel Like factor 4 (KLF4) and SET domain containing 7 (SETD7), thereby fueling the malignant proliferation of bladder cancer cells [91]. Interestingly, early-stage breast cancer cells exhibited enhanced proliferative capacity upon METTL3 silencing, while malignant transformed counterparts displayed increased migratory potential. Notably, METTL3 downregulation exerted minimal phenotypic impact on metastatic breast cancer cell lines [149]. In addition, METTL3 can methylate METTL1 mRNA and increase its expression via m^6^A in head and neck squamous cell carcinoma. Functionally, the overexpression of METTL1 increases cancer cell proliferative capacity and accelerates cell cycle advancement, whereas silencing of METTL1 suppresses these cellular processes [150]. METTL3 May posttranscriptionally upregulate the levels of inhibitor of DNA binding 2 (ID2) in an m^6^A/YTHDF2-dependent mechanism, thereby enhancing ID2 mRNA stability and promoting cancer cell proliferative capacity [151]. Furthermore, METTL16 and METTL3 are recognized as pivotal m^6^A modulators correlated with favorable prognosis in pancreatic cancer patients. Elevated METTL16 expression potently suppresses tumor progression and metastatic dissemination through repression of calpain 2 [152]. METTL14-catalyzed m^6^A modification is markedly reduced in ovarian carcinoma tissues, and reinstatement of METTL14 expression may attenuate ovarian cancer cell proliferative capacity by repressing trophinin-associated protein (TROAP) levels [153]. KIAA1429 drives breast cancer cell proliferative capacity through a non-m^6^A-dependent mechanism. This protein engages with a sequence element in the 3'-UTR of structural Maintenance of chromosomes 1A (SMC1A) mRNA, thereby enhancing its stability [154]. Specifically, KIAA1429 expression is markedly elevated in colorectal carcinoma tissues. This protein represses wee1-like protein kinase (WEE1) levels by reducing its mRNA stability through a non-m^6^A-dependent pathway, mediated by interaction with the third region in the 3'-UTR of WEE1 transcripts [70].

The impact of m^6^A demethylases on cancer cell proliferative capacity is characterized by notable heterogeneity. These enzymes, through their demethylation activity, modulate a diverse set of target transcripts, thereby eliciting varying responses in cancer cell proliferation. Specifically, ALKBH5 directly demethylates the lncRNA CARMN at 477 m^6^A sites to maintain its expression, thereby potentially suppressing mutant p53-driven breast cancer cell proliferation [155]. Moreover, low ALKBH5 expression is associated with shorter overall survival in osteosarcoma patients. ALKBH5 suppresses STAT3 signaling through elevation of suppressor of cytokine signaling 3 (SOCS3) levels, thereby inducing cell proliferative attenuation, apoptotic activation, and G1/S phase arrest [156]. FTO interacts with a conserved motif in circFAM192A, demethylating m^6^A residues to prevent endonucleolytic cleavage, thereby stabilizing the circular RNA. This stabilized circFAM192A subsequently drives gastric cancer cell proliferative capacity by inhibiting solute carrier family 7 member 5 (SLC7A5) mRNA turnover [76]. Furthermore, TF homeobox-D1 (HOXD1) and FTO form a self-reinforcing regulatory circuit that drives proliferative capacity and survival potential in head and neck cancer. FTO exhibits elevated expression in malignant tissues and transcriptionally upregulates HOXD1 levels. Conversely, HOXD1 activates the expression of the proto-oncogene FTO by directly binding to its promoter region, thereby enhancing transcription [157]. FTO enhances bladder cancer cell proliferative capacity and migratory potential by stabilizing STAT3 mRNA through an m^6^A-mediated mechanism [158]. Conversely, FTO knockdown markedly attenuates CCND1 expression in myoblasts via YTHDF2-dependent mRNA decay, arresting G1 phase progression and compromising myoblast proliferative ability [159].

**Fig. 3:**
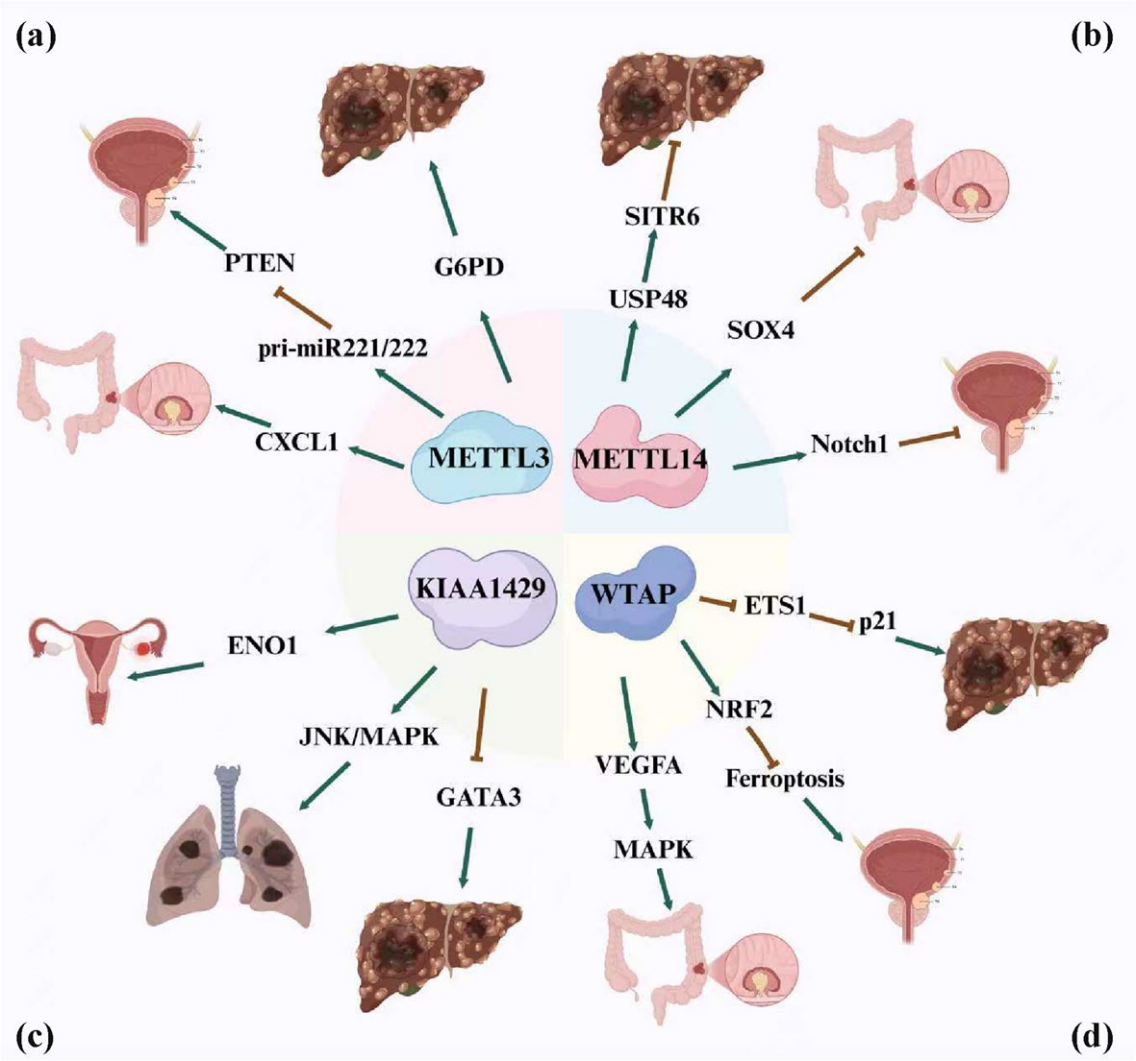
Potential mechanisms of the m^6^A writers in cancer. The m^6^A writers function as master epigenetic regulators in cancer biology. **a** METTL3 can promote colorectal cancer by upregulating CXCL1 expression. METTL3 can also promote bladder cancer by increasing pri-miR221/222 process. METTL3 might promote hepatocellular carcinoma by upregulating G6PD expression. **b** METTL14 can inhibit hepatocellular carcinoma by upregulating USP48/SITR6 axis. METTL14 can also promote colorectal cancer by upregulating SOX4 m^6^A modification. METTL14 might promote bladder cancer by upregulating Notch1 expression. **c** KIAA1429 can promote ovarian cancer by upregulating ENO1 expression. KIAA1429 can also promote lung adenocarcinoma by upregulating JNK/MAPK axis. KIAA1429 might promote hepatocellular carcinoma through downregulating GATA3 expression. **d** WTAP can promote colorectal cancer via upregulating VEGFA-MAPK axis. WTAP can also promote bladder cancer by upregulating NRF2 expression. WTAP might promote hepatocellular carcinoma through downregulating ETS1/p21 axis

The regulatory influence of m^6^A reader proteins on the proliferative capacity of malignant cells is dependent on the distinct subfamily of reader protein and the specific tumor context in which aberrant expression arises. These proteins can elicit context-dependent effects on cellular proliferation, highlighting the dynamic interplay between m^6^A-driven epigenetic modulation and the molecular heterogeneity. Specifically, YTHDF1 engages with m^6^A-decorated eIF3C mRNA and elevates their translation efficiency via a YTHDF1-mediated regulatory mechanism. This selective translational upregulation of eIF3C can promote ovarian cancer cell proliferation, which in turn accelerates tumor progression by deregulating critical checkpoints in cancer cell division [87]. Furthermore, N-acetyltransferase 10 (NAT10) interacts with YTHDF1 pre-mRNA, thereby inducing elevated skipping of YTHDF1 exon 4 that can stimulate gastric cancer cell proliferation [160]. Moreover, RNA-binding motif protein 15B (RBM15B) enhances prostate cancer cell proliferation by increasing the stability of proliferating cell nuclear antigen (PCNA) mRNA through YTHDF1-induced m^6^A methylation [161]. Furthermore, YTHDF2 exhibits elevated expression in gastric cancer cells, while AC026691.1 shows downregulated expression. The YTHDF2-dependent decay of m^6^A-modified AC026691.1 transcripts may drive proliferative capacity in gastric cancer [92]. The induction of EGFR–SRC–extracellular signal-regulated kinase (ERK) axis may stabilize YTHDF2 protein levels, which can promote the decay of LXRA and HIVEP2 mRNAs. This process contributes to cholesterol metabolic imbalance and drives enhanced tumor aggressiveness in glioblastoma cells [162]. In addition, YTHDF3 exhibits significant overexpression in breast carcinoma tissues and is linked to reduced disease-free survival rates. This protein may promote tumorigenesis and metastatic progression by interacting with eIF4B to enhance Notch2 mRNA translation efficiency in breast carcinoma [94]. Prolyl-4-hydroxylase-A2 (P4HA2) promotes papillary thyroid cancer proliferation and metastasis. YTHDF3 binding to P4HA2 decreases its stability, thereby inhibiting glycolysis [95]. Zinc finger protein 41 (ZFP41) can suppress the progression and metastasis of hepatocellular carcinoma, and YTHDF3-catalyzed m^6^A modification of ZFP41 suppresses hepatocellular carcinoma malignant progression by attenuating Snail transcriptional activation [96]. Piwi-interacting RNA 26441 (piR-26441) is aberrantly downregulated in ovarian cancer. piR-26441 suppresses mitochondrial oxidative phosphorylation and proliferative capacity in ovarian carcinoma cells via YTHDC1-mediated m^6^A modification, thereby attenuating tumor metabolic adaptation and growth [97]. β-Arrestin 2 (ARRB2) may enhance cervical cancer proliferation by stabilizing CDC25A mRNA through an IGF2BP1-dependent m^6^A regulatory mechanism [106]. IGF2BP2 promotes cyclin-dependent kinase 6 (CDK6) translation via eIF4A1 recruitment, driving cell cycle progression and G1/S phase transition in triple-negative breast cancer [112]. hnRNPA2B1-orchestrated m^6^A modification suppresses circCDYL expression, preventing its association with eIF4A3 and ultimately promoting colorectal cancer cell proliferation [117]. In conclusion, the influence of m^6^A modification on cancer cell proliferation is intricately tied to the histological subtype of the tumor and is profoundly contingent upon the deregulation of specific genes and the activation or suppression of distinct signaling pathways. The dynamic interplay among m^6^A-modifying enzymes, regulatory factors, and effector proteins orchestrates a multifaceted regulatory network that modulates gene expression profiles, ultimately shaping the proliferative behavior of cancer cells in a highly context-dependent and tumor-specific manner (Fig. 3).

#### Apoptosis

Cancer cells exhibit a fundamental survival strategy centered on evading apoptosis, driven by an imbalance between pro-apoptotic and anti-apoptotic factors that favors cellular persistence. m^6^A modification constitutes a pivotal determinant of apoptotic equilibrium, modulating the balance of pro-apoptotic/anti-apoptotic genes and the dynamic activation thresholds of apoptotic signaling cascades. Specifically, METTL3 is upregulated in hepatocellular carcinoma, and its knockdown represses cell invasion and migration while promotes apoptosis [163]. In addition, Y-box-binding protein 1 (YBX1) can interact with IGF2BPs and stabilize m^6^A-tagged RNA. YBX1 deficiency may dysregulate the expression of apoptosis-related genes and promote the decay of MYC and BCL2 mRNAs, which may contribute to decreased survival resulting from the deletion of YBX1 [164]. Moreover, YTHDF1 suppresses breast cancer apoptosis by m^6^A-mediated stabilization of aurora kinase A (AURKA) mRNA, while peptidyl-prolyl cis–trans isomerase NIMA-interacting 1 (PIN1) overexpression enhances YTHDF1 stability through inhibition of its degradation [165]. Importantly, YTHDF2 suppression induces proteotoxicity-driven cell death in MYC-overexpressing breast cancer. This protein binds MAPK pathway-related mRNAs, and its functional impairment promotes apoptosis in triple-negative breast cancer cells through disrupted mRNA decay [166]. Furthermore, YTHDC1 is increased in high-malignancy colorectal cancer tissues. YTHDC1 silencing potently suppresses colorectal cancer cell proliferation by enhancing apoptotic cell death [98]. YTHDC1 binding to m^6^A sites in ferroptosis suppressor protein 1 (FSP1) 3'-UTR results in the recruitment of the cleavage stimulatory factor subunit 3 (CSTF3) to generate a less stable FSP1 mRNA containing a shorter 3'-UTR, whereas YTHDC1 downregulation generates an FSP1 mRNA containing a longer 3'-UTR that is stabilized by the RNA-binding protein HuR and thus leads to increased FSP1 protein levels [99]. YTHDC2 induces apoptosis in papillary thyroid cancer by facilitating CYLD-dependent AKT signaling deactivation. CYLD knockdown negates YTHDC2's pro-apoptotic effects [101]. In non-small cell lung cancer, YTHDC2 reduces ID3 mRNA m^6^A levels. YTHDC2 overexpression synergizes with ID3 to markedly enhance apoptotic cell death [102]. In summary, m^6^A modification acts as a critical epigenetic hub in apoptosis, with writers and readers orchestrating gene-specific effects that dictate cancer cell fate. Deciphering these pathways may enable the development of m^6^A-targeted therapies to modulate apoptosis and increase antitumor efficacy.

#### Ferroptosis

Ferroptosis denotes a regulated cell death modality dependent on iron-driven intracellular metabolism, distinct from classical pathways such as apoptosis and necrosis [167, 168]. m^6^A modification governs ferroptotic regulation in cancer cells through epigenetic modulation of iron-dependent death pathways. Specifically, WTAP elevates Lipocalin 2 expression by enhancing nuclear protein 1 (NUPR1) m^6^A methylation, thereby repressing ferroptosis and facilitating triple-negative breast cancer progression [169]. WTAP enhances bladder cancer cell survival and suppresses erastin-driven ferroptosis by promoting methylation of a critical m^6^A site in the nuclear factor erythroid 2-related factor 2 (NRF2) mRNA 3'-UTR, which is subsequently stabilized via YTHDF1-mediated recognition of this modification [66].

**Fig. 4:**
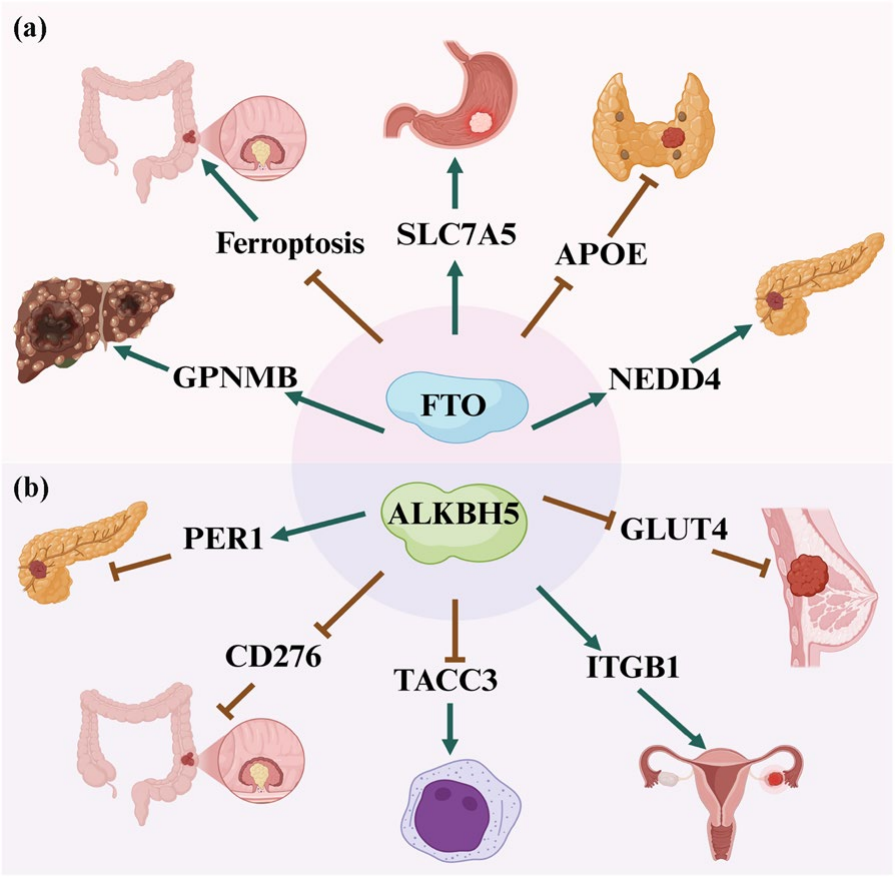
Possible mechanisms of the m^6^A erasers in cancer. The figure demonstrates the regulatory roles of the m^6^A erasers FTO and ALKBH5 in cancer. **a** FTO can promote hepatocellular carcinoma by upregulating GPNMB expression. FTO can inhibit colorectal cancer by inhibiting ferroptosis. FTO can promote gastric cancer by upregulating SLC7A5 stability. FTO may inhibit papillary thyroid cancer by downregulating APOE expression. FTO may promote pancreatic cancer by upregulating NEDD4 expression. **b** ALKBH5 can inhibit pancreatic cancer by upregulating PER1 expression. ALKBH5 can inhibit colorectal cancer via decreasing CD276 expression. ALKBH5 can promote acute myeloid leukemia through downregulating TACC3 expression. ALKBH5 might promote ovarian cancer by increasing ITGB1 expression. ALKBH5 may inhibit breast cancer by downregulating GLUT4 expression

WTAP suppression attenuates circCMTM3 m^6^A modification, thereby promoting ferroptosis in hepatocellular carcinoma, while circCMTM3 silencing destabilizes parkinsonism-associated deglycase (PARK7) via IGF2BP1 binding to induce ferroptosis [107]. Moreover, FTO overexpression upregulates solute carrier family 7 member 11 (SLC7A11) and glutathione peroxidase 4 (GPX4) expression through m^6^A-YTHDF2-dependent mechanisms, thereby suppressing ferroptosis, while FTO inhibition induces ferroptosis in colorectal cancer cells via downregulation of these anti-ferroptotic factors [77]. The lncRNA CBSLR interacts with YTHDF2, which reduces cystathionine beta-synthase (CBS) mRNA stability by enhancing YTHDF2 binding to the m^6^A-modified CDS of CBS mRNA. Upon diminished CBS expression, the methylation of the acyl-CoA synthetase long-chain family member 4 (ACSL4) protein is reduced, leading to the polyubiquitination and degradation of ACSL4 protein [170]. In hepatocellular carcinoma, ferroptosis induces a surge in m^6^A modification levels, enabling YTHDC2 to bind the m^6^A site on autophagy related gene 5 (ATG5) mRNA. This interaction enhances translational efficiency of ATG5, thereby activating autophagy-dependent ferroptosis and ultimately suppressing malignant progression [171]. Overall, m^6^A modification exerts a pivotal influence on ferroptosis through tight modulation of iron homeostasis, lipid peroxidation dynamics, and critical signaling pathways (Fig. 4).

#### Invasion and metastasis

Cancer cells exhibit a critical metastatic capability marked by their ability to breach anatomical boundaries and establish distant tumors, a process driven by epithelial–mesenchymal plasticity and microenvironmental remodeling. m^6^A modification exerts a critical influence on tumor metastasis and invasion by epigenetically regulating the expression of key metastasis-driving genes, driving EMT and organ-specific colonization. Specifically, tumor necrosis factor-alpha (TNF-α) drives bladder cancer metastasis via METTL3-mediated m^6^A modification of cytoplasmic Linker associated protein 2 (CLASP2). This modification augments CLASP2 interaction with IQ motif containing GTPase activating protein 1 (IQGAP1), thereby inducing F-actin cytoskeleton remodeling to facilitate invasive migration [172]. Elevated expression of beaded filament structural protein 1 (BFSP1) in hepatocellular carcinoma facilitates tumor invasiveness and aerobic glycolysis, with METTL3-mediated m^6^A methylation of BFSP1 mRNA enhancing its post-transcriptional stability through a YTHDF1-dependent mechanism, thereby augmenting BFSP1-driven oncogenic functions [173]. In addition, *Fusobacterium nucleatum* promotes colorectal cancer metastasis through YAP signaling pathway activation and FOXD3 transcriptional repression. METTL3 downregulation subsequently enhances kinesin family member 26B (KIF26B) expression via diminished m^6^A modification of its mRNA and attenuated YTHDF2-mediated mRNA decay, thereby facilitating oncogenic progression [174]. Furthermore, LINC00882 is upregulated in metastatic breast cancer, where it facilitates cancer cell invasion and metastatic progression. The METTL14-mediated m^6^A modification of LINC00882 may promote its expression via an IGF2BP2-dependent pathway [175]. Interestingly, METTL14 expression is also significantly increased in lung adenocarcinoma tissues, and METTL14 knockdown markedly reduces cancer cell migration and invasion. IGF2BP2 enhances glucose-6-phosphate dehydrogenase (G6PD) mRNA stability following METTL14-catalyzed m^6^A modification, thereby facilitating tumorigenesis and metastatic dissemination through metabolic reprogramming [176]. In addition, METTL16 suppression attenuates breast cancer progression by inhibiting tumor growth and metastatic dissemination. Mechanistically, METTL16-mediated m^6^A modification enhances FBXO5 mRNA stability through a post-transcriptional regulatory mechanism, thereby driving its oncogenic functions and facilitating malignant progression in breast cancer [177]. Hypoxia drives hepatocellular carcinoma metastasis through the METTL16/lncRNA CSMD1-7 axis. METTL16 interacts with lncRNA CSMD1-7 and destabilizes it via m^6^A methylation, thereby facilitating malignant progression [178]. Moreover, WTAP-mediated m^6^A modification enhances gastric cancer cell migratory and invasive capacities through upregulation of mitogen-activated protein 2 kinase 6 (MAP2K6), thereby promoting malignant progression [179]. Under hypoxia, metastasis-associated lung adenocarcinoma transcript 1 (MALAT1) upregulates WTAP expression, which modulates hypoxic response by boosting transcription of master regulators HIF1α/HIF1β. Furthermore, WTAP enhances breast cancer cell migration capacity and induces mesenchymal markers N-cadherin and vimentin [180]. Interestingly, FTO-catalyzed m^6^A modification of potassium voltage-gated channel subfamily A regulatory beta subunit 2 (KCNAB2) is implicated in non-small cell lung cancer progression. FTO depletion enhances KCNAB2 expression, thereby suppressing tumor cell migratory capacity, invasive potential, and M2 macrophage polarization [181]. FTO contributes to cervical cancer malignancy by mediating m^6^A demethylation of PIK3R3 mRNA, thereby modulating its expression and promoting oncogenic progression [182]. circGPR137B colocalizes with miR-4739 in the cytoplasm and acts as a sponge for miR-4739 to upregulate its target FTO, which mediates the m^6^A demethylation of circGPR137B and promotes its expression. The circGPR137B/miR-4739/FTO feedback loop can suppress the tumorigenesis and metastasis of hepatocellular carcinoma [183]. Additionally, ALKBH5 attenuates gastric cancer tumorigenesis and metastatic progression by repressing the translation of uncapped WRAP/53 RNA isoforms through translational regulatory mechanisms [184]. Notably, YTHDF1 interacts with m^6^A-modified sites in rho Guanine nucleotide exchange factor 2 (ARHGEF2) mRNA, thereby enhancing its translational efficiency and promoting ARHGEF2 protein synthesis. The ectopic expression of ARHGEF2 restores the impaired metastatic ability caused by the loss of YTHDF1 in colorectal cancer [88]. YTHDF2 expression is also significantly increased in oral squamous cell carcinoma tissues and cells, with its levels closely correlated with the clinical stage, pathological grade, and survival time of patients. YTHDF2 knockdown results in significant reductions in the migration and invasion abilities of cancer cells [185]. Furthermore, the ectopic overexpression of X-linked inhibitor of apoptosis protein (XIAP) promotes the degradation of YTHDC1, whereas genetic ablation of XIAP elevates YTHDC1 expression, thereby suppressing metastatic progression in bladder cancer. Mechanistically, YTHDC1 reduces MMP-2 expression by destabilizing its mRNA, establishing XIAP as a master regulator of YTHDC1. This XIAP-YTHDC1-MMP-2 regulatory axis represents a potential therapeutic target for bladder cancer intervention [186]. In addition, IGF2BP1 exhibits dysregulated overexpression in esophageal squamous cell carcinoma tissues. IGF2BP1 recognizes and stabilizes inhibin beta A (INHBA) mRNA through RNA-binding domains, thereby enhancing INHBA protein synthesis. Elevated INHBA activates SMAD2/3 signaling pathway [108]. IGF2BP2 interacts with m^6^A-modified sites in hyaluronan-mediated motility receptor (HMMR) mRNA through its RNA-binding domains, thereby enhancing mRNA stability and elevating HMMR protein expression. HMMR subsequently binds to MAP4K4, triggering phosphorylation of JNK and c-JUN, which induces MMP1 expression to promote extracellular matrix degradation and tumor metastatic dissemination [113]. HNRNPA2B1-mediated epitranscriptomic modification of Maternally expressed gene 3 (MEG3) through m^6^A methylation promotes oncogenic progression and metastatic dissemination in non-small cell lung cancer by modulating the miR-21-5p/PTEN signaling axis [118].

EMT serves as a pivotal process in cell development, marked by epithelial cells shedding their initial phenotype and adopting mesenchymal fibroblast characteristics, such as diminished cell–cell adhesion and heightened movement capability. Notably, EMT facilitates the conversion of tumor epithelial cells into mesenchymal cells capable of metastasis, granting tumor cells potent invasive properties [187–189]. These mesenchymal cells migrate to other tissues and subsequently form secondary tumors. Specifically, high METTL3 and YTHDF1 expression is detected in neoplastic tissues from colorectal cancer patients with pulmonary metastases. METTL3 drives EMT in colorectal carcinoma by epigenetically modifying Snail mRNA through m^6^A methylation. This modification enables Snail to enhance CXCL2 secretion via activation of the NF-κB signaling pathway [190]. The m^6^A modification of zinc finger MYM-type containing 1 (ZMYM1) mRNA by METTL3 increases its stability via a reader protein HuR-dependent pathway. ZMYM1 interacts with and suppresses the E-cadherin promoter, thereby driving EMT process and metastatic progression in gastric carcinoma [191]. Interestingly, homeobox A10 (HOXA10) upregulation increases m^6^A levels and METTL3 expression in gastric cancer, possibly by promoting the TGFB2/SMAD pathway. HOXA10 drives EMT to enhance metastatic progression in gastric carcinoma, partially through regulation of the TGFB2/SMAD/METTL3 signaling cascade [192]. Moreover, METTL14 can stabilize ubiquitin specific peptidase 38 (USP38) mRNA by inducing m^6^A modification. METTL14 can suppress bladder cancer EMT through USP38. Additionally, miR-3165 can inhibit METTL14 expression to promote bladder cancer progression. The reciprocal regulatory circuit between METTL14 and USP38 can promote EMT in bladder cancer [193]. Elevated m^6^A levels in papillary and anaplastic thyroid cancer are counteracted by FTO restoration which suppresses invasion and metastasis via EMT modulation. Meanwhile, IGF2BP2 stabilizes cadherin 12 (CDH12) mRNA to drive EMT progression and metastatic aggressiveness [114]. Furthermore, follicle-stimulating hormone (FSH) promotes EMT progression by prolonging the half-life of Snail mRNA in ovarian cancer [194]. Notably, YTHDC1 is vital for triple-negative breast cancer progression because it increases cancer cell survival and TGF-β-mediated EMT via SMAD3 to promote distant metastasis, highlighting the therapeutic potential of targeting the YTHDC1–m^6^A–SMAD3 axis [195].

**Fig. 5:**
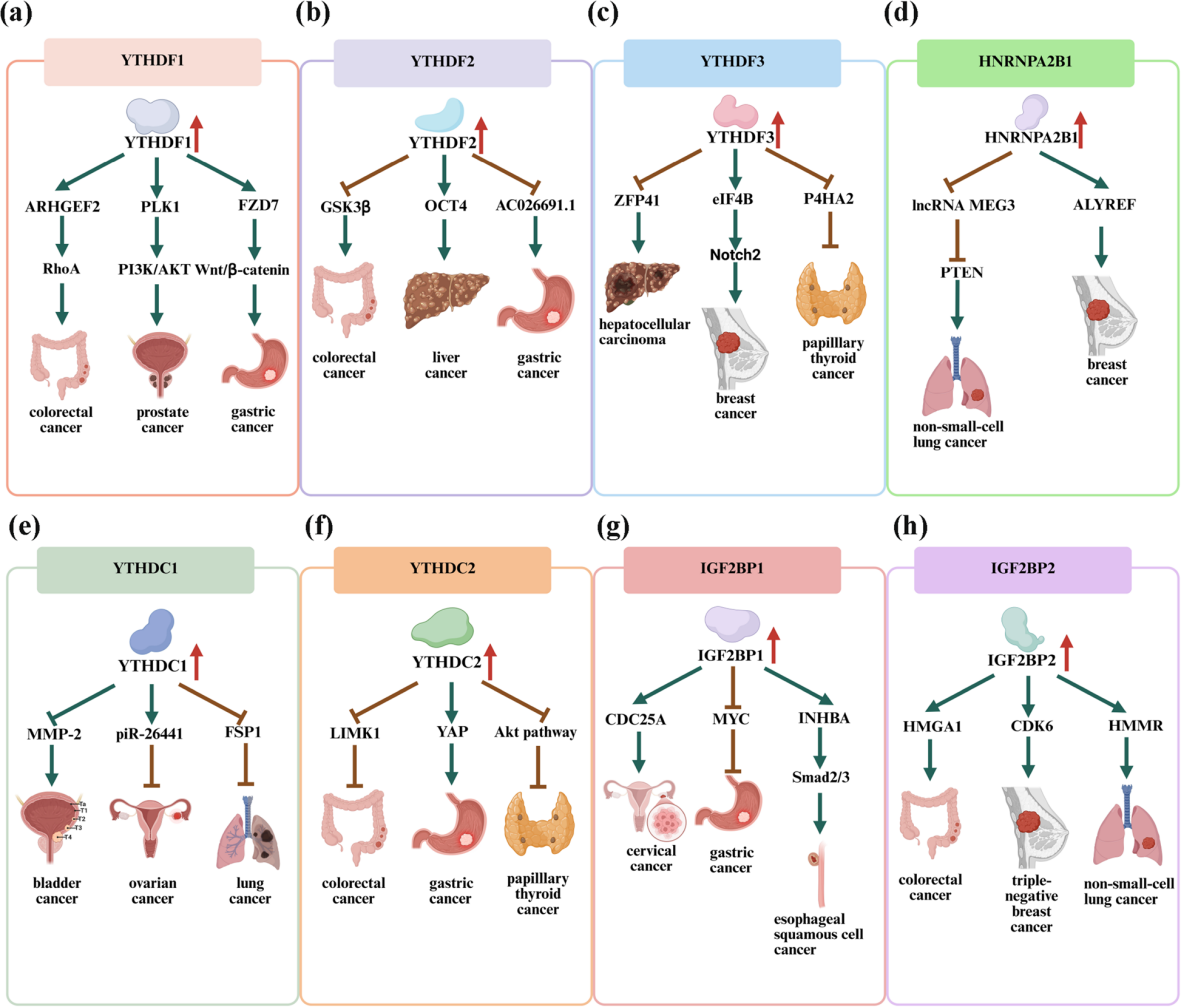
Underlying mechanisms of the m^6^A readers in cancer. This figure indicates the possible regulatory mechanism of m^6^A readers in cancer. **a** YTHDF1 can promote colorectal cancer by upregulating ARHGEF2/RhoA signaling. YTHDF1 may promote prostate cancer by upregulating PLK1–PI3K–AKT axis. YTHDF1 might promote gastric cancer by upregulating FZD7-Wnt/β-catenin. **b** YTHDF2 can promote colorectal cancer by downregulating GSK3β expression. YTHDF2 may promote liver cancer by downregulating OCT4 expression. YTHDF2 might promote gastric cancer by downregulating Ac026691.1 expression. **c** YTHDF3 can promote hepatocellular carcinoma by downregulating ZFP41 expression. YTHDF3 might promote breast cancer by upregulating eIF4Bt-Notch2 axis. YTHDF3 may inhibit papillary thyroid cancer by downregulating P4HA2 expression. **d** HNRNPA2B1 can promote non-small cell lung cancer by downregulating lncRNA MEG3/PTEN axis. HNRNPA2B1 may promote breast cancer by upregulating ALYREF expression. **e** YTHDC1 can promote bladder cancer by downregulating MMP-2 expression. YTHDC1 may inhibit colorectal cancer by upregulating piR-26441 expression. YTHDC1 might inhibit lung cancer through downregulating FSP1 expression. **f** YTHDC2 can inhibit colorectal cancer by downregulating LIMK1 expression. YTHDC2 may promote gastric cancer by upregulating YAP expression. YTHDC2 might inhibit papillary thyroid cancer via downregulating Akt pathway. **g** IGF2BP1 can promote cervical cancer through upregulating CDC25A expression. IGF2BP1 may inhibit gastric cancer by downregulating MYC expression. IGF2BP1 might promote esophageal squamous cancer by upregulating INHBA/Smad2/3 axis. **h** IGF2BP2 can promote colorectal cancer by upregulating HMGA1 expression. IGF2BP2 may promote triple-negative breast cancer by upregulating CDK6 expression. IGF2BP2 might promote non-small-cell lung cancer by upregulating HMMR expression

Migrating tumor cells can break through tissue barriers and form metastases in distant organs. METTL3 drives tumorigenesis and lung/lymph node metastasis in gastric cancer via the PBX1/GCH1 axis, elevating tetrahydrobiopterin levels to enhance metastatic aggressiveness [196]. Importantly, KIAA1429 enhances lung metastatic potential in hepatoma cells by targeting GATA3 pre-mRNA through m^6^A modification of its 3'-UTR, triggering HuR dissociation and subsequent degradation. This process is facilitated by the cis-acting lncRNA GATA3-AS, which promotes preferential KIAA1429–GATA3 pre-mRNA interaction [71]. Moreover, ALKBH5 upregulation counteracts m^6^A modification of integrin beta-1 (ITGB1) mRNA, inhibiting m^6^A-mediated decay and elevating ITGB1 levels. This upregulates the focal adhesion kinase (FAK) and Src proto-oncogene protein phosphorylation, thereby enhancing lymph node metastatic dissemination in ovarian cancer [81]. Importantly, upregulated YTHDF1 associates with bone metastasis in breast cancer by promoting migration and invasion, inducing osteoclast differentiation, and enhancing Zeste Homolog 2 (EZH2) translation to drive osteolytic progression [89]. YTHDC2 enhances YAP translation efficiency by recognizing m^6^A-modified YAP mRNA at the 5’-UTR without altering mRNA levels, while YAP directly activates YTHDC2 transcription via promoter binding, forming a positive feedback loop. YTHDC2 knockout significantly reduces tumor size and lung metastatic nodules in gastric cancer [103]. Future research priorities for m^6^A epitranscriptomic regulation in cancer metastasis and invasion include exploring its role in organ-specific metastasis, developing m^6^A-targeted therapies, and elucidating interactions with immune cells and survival pressures (Fig. 5).

#### Angiogenesis

Angiogenesis plays a role in numerous biological activities and is involved in various pathological phenomena. In the context of tumor progression, angiogenesis serves as a critical determinant of invasive and metastatic potential, driven by the overexpression of proangiogenic factors such as vascular endothelial growth factor A (VEGFA) in endothelial cells [197]. Moreover, m^6^A regulation is extensively involved in tumor angiogenesis. Modulating the structural modifications and physiological processes of VEGFA through m^6^A modification can influence angiogenesis. Notably, METTL3 decreases VEGFA production via the m^6^A reader IGF2BP3, inhibiting gastrointestinal tumor-associated angiogenesis [198]. Moreover, ALKBH5 attenuates m^6^A levels within the 3'-UTR and CDS regions of zinc finger protein with KRAB and SCAN domains 3 (ZKSCAN3) mRNA, thereby facilitating its binding to the VEGFA promoter region, which ultimately contributes to migration, invasion, spheroid formation, and progression in gastric cancer [199]. In studies on hepatocellular carcinoma, YTHDF2 acts in an m^6^A-dependent Manner on its downstream direct target ETS variant transcription factor 5 (ETV5), regulating ETV5 mRNA translation to negatively modulate its protein levels and thereby influence VEGFA production [200]. In addition to regulating proangiogenic factors, m^6^A modification and its associated factors participate in disease treatment through other mechanisms. METTL3 reduces the stability of janus kinase 2 (JAK2) mRNA via the reader IGF2BP1 in human umbilical vein endothelial cells, thereby inhibiting the JAK2/STAT3 signaling pathway and suppressing oxidized low-density lipoprotein-induced angiogenesis, which holds therapeutic significance for atherosclerosis [201]. In vitro tube formation assays have demonstrated that ZNRD1-AS1 is essential for lung cancer cell proliferation and angiogenesis. YTHDC2 targets miR-942 downstream from ZNRD1-AS1 through m^6^A-mediated regulation, reducing its stability and offering therapeutic potential for lung cancer proliferation and invasion [202]. In summary, the relationship between m^6^A modification and angiogenesis is implicated in various cancer treatment strategies involving diverse regulatory factors and pathways, thereby providing novel therapeutic targets for cancer therapy.

#### Immune evasion

Tumor immune evasion represents a significant barrier to effective cancer therapy, with emerging evidence indicating that m^6^A methylation and its modifiers facilitate the establishment of an immune-suppressive niche across multiple Malignancies through reprogramming of cellular metabolism, suppression of T cell activity, and modulation of checkpoint pathways. Poor T-lymphocyte infiltration is considered a Key mechanism of tumor immune evasion, and Keratin 17 (KRT17) can increase T-lymphocyte infiltration to reverse the tumor immunosuppressive microenvironment. KRT17 is upregulated in mismatch repair-deficient colorectal cancer, where it enhances T cell infiltration via the YTHDF2/CXCL10 signaling axis, thereby reversing immune evasion [203]. YTHDF1 suppresses antitumor immune responses via the m^6^A–p65–CXCL1/CXCR2 axis, driving colorectal cancer progression and representing a potential therapeutic target for immune checkpoint inhibitor therapy [204]. Tumor-intrinsic METTL3 suppresses CD8^+^ T cell activation and effector functions, thereby driving immune evasion and non-alcoholic fatty liver disease related hepatocellular carcinoma progression. Additionally, anti-CD8a therapy compromises CD8^+^ T cell-mediated antitumor immunity and diminishes immunotherapy efficacy [205]. In colorectal cancer, circQSOX1 undergoes METTL3-mediated m^6^A methylation and exhibits enhanced stability upon IGF2BP2 binding. The m^6^A-modified circQSOX1 sequesters miR-326 and miR-330-5p, thereby upregulating phosphoglycerate mutase 1 (PGAM1) expression, which facilitates immune evasion and diminishes anti-CTLA-4 therapy efficacy. Combined treatment with sh-circQSOX1 and anti-CTLA-4 represents a promising strategy to overcome immunotherapy resistance [206]. Dysregulation of intracellular Fe^2+^/Fe^3+^ homeostasis inactivates the demethylases FTO/ALKBH5, thereby increasing cellular m^6^A levels. This increase in m^6^A destabilizes glucose transporter 3 (GLUT3)/pyruvate kinase M (PKM) transcripts that mediate glycolysis and immune checkpoint signaling, thereby suppressing cell proliferation and synergizing with PD-L1 checkpoint blockade therapy [206]. Interestingly, protein arginine methyltransferase 5 (PRMT5) directly mediates symmetric demethylation of ALKBH5 at residue R316, thereby promoting its ubiquitin–proteasome degradation. Reduced ALKBH5 levels diminish m^6^A demethylation within the 3'-UTR of CD276 mRNA, enhancing its stability and facilitating immune evasion in colorectal cancer [82]. The dysregulated overexpression of serine hydroxy methyltransferase 2 (SHMT2) in endometrial carcinoma and its prognostic significance were validated through integrated bioinformatic analysis of multiomic databases coupled with retrospective clinical cohort evaluation. Mechanistically, in vitro functional assays demonstrated that SHMT2 modulates c-MYC transcriptional output through an m^6^A-dependent epitranscriptomic mechanism, thereby orchestrating malignant cellular behaviors, including proliferative capacity, migratory/invasive phenotypes, and immunoreactive properties. Notably, SHMT2-orchestrated metabolic reprogramming has been demonstrated to directly modulate the stability of MYC mRNA via alterations in m^6^A modification patterns, as evidenced by in vivo xenograft studies in which enforced SHMT2 expression promoted tumorigenic progression through epigenetic reprogramming of proto-oncogenic networks [207]. Overall, m^6^A modification modulates immune evasion in cancer by altering immune checkpoint expression, antigen presentation, and cytokine signaling, thereby reshaping TME interactions and influencing responses to immunotherapy.

### m^6^A modification and cancer stem cells

Cancer stem cells constitute a distinct subpopulation with stemness maintenance and pluripotent differentiation potential and have emerged as essential regulators of malignant progression and treatment resistance. Emerging evidence underscores the necessity of developing targeted interventions against cancer stem cells to impede oncogenic processes and enhance therapeutic efficacy [208]. Among the epigenetic regulatory mechanisms, m^6^A modification serves as a crucial mediator in cancer stem cell biology. This dynamic modification not only participates in cancer stem cell generation and maintenance but also modulates tumor aggressiveness and resistance mechanisms through the posttranscriptional regulation of oncogenic pathways [209]. METTL3 shows up-regulated expression in glioma stem-like cells versus their differentiated counterparts, with its expression progressively attenuating during lineage commitment. Notably, METTL3 depletion results in heightened radiosensitivity accompanied by impaired DNA damage response efficiency [210]. METTL14 facilitates m^6^A modification and promotes the degradation of activating transcription factor 5 (ATF5) mRNA. Upregulation of ATF5 reverses the stemness attenuation induced by METTL14 upregulation through enhancing WD repeat domain 74 (WDR74) transcription and increasing β-catenin nuclear translocation [211]. Interestingly, METTL14 is expressed at low levels in bladder cancer, and METTL14 knockout can promote proliferation, self-renewal, metastasis and tumor-initiating capacity. METTL14 can inhibit the self-renewal of tumor-initiating cells and bladder tumorigenesis through m^6^A modification of Notch1 [63]. METTL16 exhibits elevated expression in liver cancer stem cell, with its depletion markedly attenuating the population of cancer stem cells. METTL16 can promote self-renewal capacity by upregulating ribosome biogenesis and translational process activation [212]. Furthermore, WTAP and m^6^A levels are decreased in endometrial cancer stem cells, and WTAP knockdown promotes properties of endometrial cancer stem cells, encompassing proliferative potential, invasive ability, migratory capacity, resistance to cisplatin, and self-renewal capacity. WTAP knockdown attenuates m^6^A methylation of EGR1 mRNA, impairing IGF2BP3-mediated recognition and binding, which subsequently reduces EGR1 mRNA stability [213]. In addition, ALKBH5 is essential for the initiation and progression of acute myeloid leukemia and the stemness maintenance of leukemia stem cells, yet it is dispensable for normal hematopoiesis. Mechanistically, ALKBH5 may exert pro-tumorigenic activities on acute myeloid leukemia via the posttranscriptional regulation of its critical targets [83]. ALKBH5 can demethylate inosine triphosphatase mRNA resulting in elevated stability. The transcription factor 15 (TCF15), which is specifically expressed in leukemia stem cells, is responsible for the dysregulated expression of ALKBH5 in acute myeloid leukemia [214]. Interestingly, FTO levels are reduced in ovarian stem cells, whereas FTO reintroduction attenuates stemness maintenance in these cells. By reducing m^6^A levels at the 3'-UTR and the mRNA stability of two phosphodiesterase genes, FTO increases cAMP signaling and suppresses the stem-like properties in ovarian carcinoma cells [215]. Notably, YTHDF1 promotes liver cancer stemness maintenance coupled with tyrosine kinase inhibitors resistance (lenvatinib, sorafenib) within patient-derived organoid models. YTHDF1 binds to the m^6^A-methylated Notch1 mRNA exhibits increased stability and translational activity, leading to upregulation of downstream Notch1 target genes [216]. Consistently, the elevated YTHDF1 protein is detected in human acute myelogenous leukemia, and is concentrated in leukemia stem cells. YTHDF1 knockdown suppresses stemness maintenance, proliferative capacity, and leukemia-initiating ability in primary human/mouse leukemia stem cells [217]. In addition, YTHDF2 can maintain oncogene MYC and VEGFA transcripts in glioblastoma stem cells, thereby offering a therapeutic opportunity in glioblastoma [218]. YTHDF2 expression is inversely associated with overall survival in hepatocellular carcinoma patients, indicating a poor prognostic signature, and the knockdown of YTHDF2 leads to impaired stemness. YTHDF2 drives the cancer stem cell phenotype and metastatic progression in Liver cancer by augmenting octamer-binding transcription factor 4 (OCT4) expression via m^6^A methylation [219]. The YAP1 knock-down in bladder cancer stem cells can impede the YTHDF3-mediated degradation of m^6^A-modified SMAD7, culminating in decreased cell stemness [220]. Additionally, YTHDC1 expression is robustly induced in leukemic stem cells, which may be correlated with adverse clinical outcomes in acute myeloid leukemia patient. Elevated YTHDC1 expression functions as an oncogenic driver in leukemia by enhancing stem cell self-renewal, inhibiting apoptosis, and consequently increasing the reservoir of leukemic stem cells in the hematopoietic niche [221]. Interestingly, increased IGF2BP1 expression has been confirmed in non-small cell lung cancer tissues and cancer stem cells. IGF2BP1 knockdown suppresses the stemness maintenance, self-renewal capacity, xenograft tumorigenesis, and immune evasion of cancer stem cells [109]. Elevated IGF2BP2 expression in esophageal cancer tissues correlates with aggressive tumor behavior, as IGF2BP2 overexpression potentiates stem-like traits in cancer cells. This occurs through IGF2BP2-mediated recognition of m^6^A-modified OCT4 mRNA, which increases OCT4 mRNA stability and protein levels, ultimately driving stemness and tumorigenic potential [93]. hnRNPA2B1 selectively facilitates the nuclear export of m^6^A-modified mRNAs by interacting with the Aly/REF export factor (ALYREF)-nuclear RNA export factor 1 (NXF1) complex, thereby enhancing the expression of stemness-related genes critical for cancer stem cell maintenance [119]. Overall, m^6^A modification has emerged as a pivotal epigenetic determinant that governs the biological characteristics of cancer stem cells, including stemness maintenance, differentiation, dynamic regulation of self-renewal, and chemoresistance acquisition. By orchestrating the posttranscriptional regulation of specific gene networks, m^6^A-dependent mechanisms create a permissive molecular environment that sustains cancer stem cell plasticity and therapeutic recalcitrance.

### m^6^A modification and TME

The TME comprises a stratified heterocellular network that integrates malignant cells, immune cells, stromal fibroblasts, angiogenic vasculature, extracellular matrix (ECM), and paracrine signaling molecules. This dynamic milieu profoundly influences oncogenic initiation, malignant progression, metastatic dissemination, and therapeutic efficacy. This review synthesizes the pathophysiological roles of malignant cells, immune cells and stromal components in the TME, emphasizing their synergistic contributions to immunosuppression, metabolic reprogramming, and paracrine signaling that drive cancer progression and therapeutic resistance [222–224].

#### Malignant cells

Malignant cells are central to the TME, driving its structural and functional remodeling. They orchestrate metabolic reprogramming, angiogenesis, and immunosuppression through secreted factors and extracellular interactions [222]. Notably, m^6^A methylation is vital in malignant cell survival by epigenetically controlling mRNA the stability of and genes translation [223]. Dysregulated m^6^A dynamics alter malignant cell behavior, influencing tumor progression and TME adaptation [224]. This modification thus represents a key layer of post-transcriptional control essential for Maintaining the Malignant phenotype and microenvironmental balance. Specifically, 5-fluorouracil (5-FU)-resistant colorectal cancer cells exhibit enhanced ATP generation, glucose consumption, lactate production, and oxygen consumption rates, reflecting metabolic adaptations to the TME. Elevated m^6^A modification and METTL3 expression in these cells drive glycolysis, while METTL3 inhibition or knockdown suppresses glycolysis and restores chemosensitivity, highlighting a critical link between m^6^A-mediated metabolic reprogramming and TME-driven drug resistance [225]. Moreover, hepatitis B virus X-interacting protein (HBXIP) is elevated expression in hepatocellular carcinoma tissues under hypoxic TME conditions. METTL3, positively regulated by HBXIP, is similarly elevated and drives metabolic reprogramming and malignant progression. In the hypoxic TME, HBXIP-activated METTL3 binds to HIF-1α, facilitating its m^6^A modification to enhance hypoxia-adaptive metabolic reprogramming and aggressive tumor behaviors [226]. Interestingly, USP48 stabilizes SIRT6 via K48-linked deubiquitination at K33/K128 residues, suppressing metabolic reprogramming and reducing lactate/acidic metabolite accumulation that acidifies the TME and promotes immunosuppression. METTL14-mediated m^6^A modification stabilizes USP48 mRNA, linking epigenetic regulation to TME metabolic remodeling and hepatocellular carcinoma suppression [64]. Besides, KIAA1429 promotes tumor progression and glycolysis, which elevates lactate levels and acidifies the TME, fostering immunosuppression and malignant progression by stabilizing ENO1 mRNA. SPI1, the key transcription factor regulating KIAA1429, forms a SPI1–KIAA1429–ENO1 axis that drives metabolic reprogramming, connecting m^6^A-mediated epigenetic changes to TME remodeling and ovarian cancer aggressiveness [72]. Furthermore, METTL3 upregulates basic helix-loop-helix family member E41 (BHLHE41) expression, which subsequently induces CXCL1 transcription in the inflammatory TME, enriching inflammatory factors and recruiting myeloid-derived suppressor cells (MDSCs) to suppress tumor-specific immune response. By targeting the BHLHE41–CXCL1/CXCR2 signaling axis, METTL3 promotes colorectal cancer progression through remodeling of the inflammatory TME. [59]. In addition, glycoprotein nonmetastatic melanoma protein B (GPNMB), a downstream target of FTO that reduces its m^6^A abundance, is packaged into small extracellular vesicles (sEVs) derived from hepatocellular carcinoma cells. These sEVs deliver GPNMB to the TME, where it binds syndecan-4 on CD8^+^ T cells, suppressing their activation and fostering an immunosuppressive TME [78]. In the breast cancer immune microenvironment, PD-L1 functions as a direct target of METTL3-regulated m^6^A epigenetic modification. METTL3 knockdown reduces PD-L1 m^6^A levels, thereby attenuating its inhibitory effect on T cell activation and augmenting antitumor immunity through reinvigoration of cytotoxic T cells [227]. Importantly, WTAP upregulates PD-L1 expression through m^6^A-dependent mechanisms in the tumor immune microenvironment, while IGF2BP2 stabilizes the methylated PD-L1 transcript, reinforcing its accumulation and immunosuppression. This WTAP–IGF2BP2–PD-L1 axis suppresses T cell proliferation and cytotoxic activity [228]. Overall, m^6^A modification in malignant cells reshapes the TME via metabolic reprogramming and extracellular vesicle-mediated signaling, fostering a pro-tumor niche. These dynamic influences therapy resistance by altering cell–cell communication and metabolic dependencies, highlighting m^6^A regulators as potential targets to disrupt tumor-microenvironment crosstalk and improve treatment outcomes.

#### Immune cells

Immune cells within the TME exhibit a dual regulatory role where cytotoxic subsets directly eliminate malignant cells while immunosuppressive populations drive tumor immune escape. m^6^A modification acts as a pivotal epigenetic switch by modulating transcripts critical for immune cell activation and cytokine signaling. This dynamic creates an immunosuppressive niche through reprogrammed immune interactions, balancing antitumor immunity and pro-tumor tolerance. Therapeutically, targeting m^6^A pathways could disrupt this equilibrium, reactivate immune surveillance and enhancing immunotherapy efficacy. Specifically, macrophage-specific METTL14 deficiency can trigger significant functional impairment in tumor-infiltrating CD8^+^ T cells, characterized by compromised effector cytokine production and the marked upregulation of exhaustion-associated checkpoints. Consequently, defective CD8^+^ T cell trafficking into the tumor parenchyma is accompanied by a diminished cytotoxic capacity, which collectively promotes colorectal cancer progression by establishing immunosuppressive reprogramming [229]. Moreover, the deletion of METTL3 or METTL14 increases the infiltration of cytotoxic CD8^+^ T cells and the secretion of the cytokines IFN-γ, CXCL9, and CXCL10, thereby improving the response to anti-PD-1 therapy in colorectal cancer [230]. Interestingly, the copiousness of mRNA m^6^A is positively associated with natural killer (NK) cell activation and antitumor functions of metastatic melanoma. Blocking the activation of mTOR complex 1 suppresses m^6^A methylation levels during the activation of NK cells, and this suppression can be reversed through S-adenosylmethionine supplementation [231]. Furthermore, SMAD4 overexpression enhances NKG2D activation via YTHDF2 upregulation, thereby potentiating NK cell cytotoxicity against colorectal cancer cells through the SMAD4/YTHDF2 regulatory axis [232]. Besides, m^6^A-dependent epigenetic modulation promotes miR-146b biogenesis and processing, while concurrently inducing polarization of tumor-associated macrophages toward an immunosuppressive M2 phenotype. Consequently, this axis suppresses immune cell infiltration into the colorectal cancer microenvironment and facilitates adaptive immune evasion mechanisms [233]. In addition, USP14 can enhance CXCL2 expression by stabilizing the m^6^A reader IGF2BP2 in tumor-associated macrophages. The USP14–IGF2BP2–CXCL2 axis in macrophages significantly promotes the therapeutic sensitivity of gastric cancer to anti-PD-1 therapy [234]. Attractively, IL-1β and TNFα secreted by C5aR1-positive neutrophils work in tandem to trigger ERK1/2 signaling. This activation is Likely to lead to the phosphorylation of WTAP at serine 341, resulting in the stabilization of the WTAP protein. Once stabilized, WTAP can boost the m^6^A methylation of ENO1 mRNA, which in turn increases glycolytic activity and drives heightened aggressiveness in breast cancer cells [67]. METTL3 deficiency enhances IL-8 production in papillary thyroid carcinoma cells, thereby promoting the recruitment of tumor-associated neutrophils. This neutrophilic infiltration subsequently drives cancer progression through the pro-tumorigenic activities of neutrophils within the TME [60]. Importantly, YTHDF1 within dendritic cells is capable of recognizing m^6^A-modified transcripts of lysosomal proteases, and its knockout abolishes the tumor antigen cross-presentation capacity of classical dendritic cells. Consequently, this molecular perturbation promotes CD4^+^/CD8^+^ T cell immune infiltration and suppresses gastric cancer progression through reactivated antitumor immunity [235]. Additionally, YTHDF2 bridges m^6^A-dependent degradation of bone morphogenetic protein and activin membrane-bound inhibitor (BAMBI) transcripts in myeloid-derived suppressor cells (MDSCs) through NF-κB signaling, thereby coupling m^6^A modification to MDSC immunosuppressive function. Reduced BAMBI expression enhances MDSC tumor infiltration and T cell suppression, driving radioresistance and metastasis in breast cancer, while AAV-mediated BAMBI restoration synergizes with radiotherapy to improve local control and suppress distant tumors [236]. Remarkably, USP47 functions to inhibit the ubiquitination of YTHDF1, which subsequently leads to a decrease in its interaction with translation initiation complexes. This, in turn, suppresses the m^6^A—mediated translation efficiency of c-Myc. Such suppression is of paramount importance for maintaining the metabolic and functional homeostasis of regulatory T cells (Tregs). USP47 ablation destabilizes Tregs, amplifies antitumor immune responses, and accelerates glycolytic reprogramming in thyroid cancers through c-Myc-driven metabolic rewiring [237]. METTL3-mediated m^6^A modification promotes CD70 mRNA decay, limiting Treg and exhausted T cell accumulation in the TME. M2 macrophage EVs reduce METTL3 in advanced thyroid cancer via miR-21-5p, linking to anti-PD-1 resistance reversed by CD70 blockade, thus providing a strategy to overcome checkpoint inhibitor resistance [238]. Overall, m^6^A modification dynamically modulates immune cell functions, critically influencing immunotherapy efficacy. By regulating key gene expression, m^6^A reshapes immune cell composition and activity, offering therapeutic targets to enhance checkpoint inhibitor responses and overcome treatment resistance.

#### Stromal components

Stromal constituents, comprising ECM components and cancer-associated fibroblasts (CAFs), exert pivotal regulatory roles in orchestrating tumor progression dynamics and modulating therapeutic responsiveness in the tumor microenvironment. They provide structural support, secrete pro-tumorigenic factors, and remodel the ECM to create immunosuppressive niches. m^6^A modification regulates stromal dynamics by modulating key genes, influencing CAF activation and ECM stiffness. Dysregulated m^6^A in stromal components contributes to immune checkpoint inhibitor resistance. Targeting m^6^A pathways in stromal components offers a strategy to disrupt immunosuppressive networks and enhance cancer therapy efficacy. Specifically, METTL3-catalyzed m^6^A modification augments TGF-β1 transcript stability and translational efficiency by facilitating post-transcriptional mRNA processing, leading to increased secretion of TGF-β1 by osteosarcoma cells. This secreted TGF-β1 then activates signaling pathways in mesenchymal stem cells to drive their differentiation into CAFs. The METTL3/TGF-β1 signaling axis drives osteosarcoma tumorigenesis by enhancing CAFs differentiation, offering a novel therapeutic target to combat advanced metastatic disease [239]. Similarly, CAFs secrete VEGFA to elevate METTL3 expression levels in non-small cell lung cancer cells, which subsequently facilitates m^6^A methylation of ras-related C3 botulinum toxin substrate 3 (RAC3) mRNA. This post-transcriptional modification augments transcript stability and translational efficiency through AKT/NF-κB signaling pathway activation. This CAF–METTL3–RAC3 axis significantly promotes cancer cell metastasis and tumor growth, with METTL3 overexpression correlating with poor patient prognosis, highlighting its potential as a therapeutic target for metastatic lung cancer [240]. Furthermore, the m^6^A demethylase FTO is specifically upregulated in CAFs of conjunctival melanoma, where it removes m^6^A modifications from VEGFA and EGR1 mRNAs, thereby blocking YTHDF2-mediated mRNA decay and enhancing their stability and expression. This FTO-driven activation of proangiogenic CAFs significantly promotes tumor progression by fueling angiogenesis in conjunctival melanoma, thereby establishing FTO as a therapeutic target with translational potential for antiangiogenic strategies in oncology [241]. Interestingly, METTL3 within CAFs orchestrates IL-18 expression through m^6^A-mediated mRNA modification, thereby triggering NF-κB signaling to modulate PD-L1-dependent immunosuppression in non-small cell lung cancer. CAF-derived METTL3 exacerbates PD-L1-driven immune evasion, while METTL3-mediated IL-18/NF-κB signaling alleviates immunosuppression [242]. Collectively, m^6^A modification serves as a critical epigenetic regulator of stromal components within the TME, dynamically influencing their functional states. By modulating mRNA stability, translation, or decay, m^6^A enzymes alter the expression of key signaling molecules in stromal cells, thereby reprogramming their secretory profiles and activation status. This regulation shapes the TME by controlling the production of pro-tumorigenic factors and immunosuppressive mediators, which in turn affect tumor progression, metastasis, and therapy resistance. Therapeutically, targeting m^6^A pathways offers a novel strategy to disrupt stromal-tumor crosstalk. Inhibiting m^6^A enzymes could reverse stromal-mediated immunosuppression or angiogenesis, synergizing with immunotherapy or anti-angiogenic agents to enhance treatment efficacy. Understanding m^6^A-driven stromal reprogramming provides a paradigm shift in optimizing TME-targeted therapies for precision oncology.

## Implications of m^6^A modification dysregulation in cancer

m^6^A modification is emerging as a multifaceted regulator in cancer, dynamically shaping tumorigenesis, immune evasion, and therapeutic resistance. Dysregulation of m^6^A machinery not only drives oncogenic progression but also holds transformative clinical potential. Critically, aberrant m^6^A enzyme profiles serve as diagnostic biomarkers for early detection via liquid biopsies, whereas dynamic modification patterns in oncogenes provide robust prognostic stratification. m^6^A-mediated adaptive mechanisms such as metabolic reprogramming and drug-metabolizing enzyme regulation underpin treatment resistance, necessitating novel therapeutic strategies targeting m^6^A modification.

### Diagnostic potential of m^6^A modification

The epitranscriptomic machinery governing m^6^A modification has considerable diagnostic utility in oncology, as these RNA-modifying enzymes function as critical regulators of neoplastic transformation through precise control of oncogenic and tumor-suppressor transcriptional activity and production. By dynamically modulating RNA processing, stability, and translation, m^6^A-related enzymatic complexes establish posttranscriptional regulatory nodes that underpin molecular pathways that are central to tumor initiation and maintenance. These mechanisms not only reveal actionable vulnerabilities but also provide a mechanistic rationale for developing epitranscriptomic biomarkers capable of stratifying molecular subtypes, predicting therapeutic response patterns, and identifying synthetic lethal interactions in precision oncology paradigms. Specifically, non-small cell lung cancer patients exhibit elevated m^6^A methylation in leukocytes, with peripheral blood RNA profiles showing significantly increased m^6^A levels. These findings indicate the potential of circulating m^6^A signatures as noninvasive biomarkers for non-small cell lung cancer surveillance and diagnosis [243]. Additionally, elevated peripheral blood m^6^A signatures in colorectal cancer demonstrate better diagnostic performance than traditional biomarkers. Tumor-derived soluble factors may induce immune-cell m^6^A reprogramming, creating a systemic epigenetic signature detectable through liquid biopsy. Integrating m^6^A profiling with conventional biomarkers enables earlier colorectal cancer detection by capturing tumor-driven epigenetic alterations in circulating immune cells [243]. Moreover, m^6^A-related lncRNAs have emerged as clinically relevant predictive biomarkers in cancer, with 262 m^6^A-related lncRNAs identified in lung adenocarcinoma and 6 m^6^A-related lncRNAs in breast cancer demonstrating significant diagnostic potential [243]. In addition, the quantitative expression profiles of m^6^A epigenetic regulators demonstrate significant potential as noninvasive diagnostic biomarkers for malignant tumors, as evidenced by their differential abundance patterns in clinical specimens. METTL3 emerges as a clinically validated prognostic and immunoregulatory biomarker in breast cancer, whereas METTL14 is correlated with both survival outcomes and tumor-infiltrating lymphocyte dynamics in rectal adenocarcinoma [243]. Notably, FTO overexpression in gastric cancer is correlated with aggressive tumor phenotypes, including poor histological differentiation, lymphovascular invasion, advanced TNM stage, and advanced clinical stage, collectively underscoring its potential as a critical prognostic biomarker for disease progression and metastatic risk stratification [244]. FTO overexpression in breast cancer is implicated in promoting tumor aggressiveness, whereas epitranscriptomic profiling of peripheral blood RNA has identified m^6^A modifications as novel noninvasive diagnostic biomarkers. Reciprocal changes in FTO mRNA and oncogenic transcripts may synergistically increase m^6^A methylation levels, supporting the development of integrated biomarker panels that combine epigenetic and transcriptomic data for enhanced diagnostic precision [245, 246]. Furthermore, ALKBH5 downregulation is frequently observed in esophageal squamous cell carcinoma and is significantly associated with tumor progression and aggressive clinicopathological features, including advanced stage and metastatic potential [247]. Interestingly, WTAP overexpression in colorectal cancer enhances malignant phenotypes through the upregulation of cell proliferation, migratory capacity, invasive potential, and tumor angiogenesis, thereby establishing WTAP as a promising diagnostic/prognostic biomarker. Mechanistically, WTAP promotes VEGFA expression via a YTHDC1-dependent RNA splicing mechanism and activates the MAPK signaling cascade, which synergistically drives tumor angiogenesis and metastatic progression, underscoring its critical role in colorectal cancer oncogenesis [68]. In addition, WTAP expression also serves as an independent prognostic biomarker for hepatocellular carcinoma, with elevated levels correlating with poor survival outcomes. Mechanistically, WTAP functions as an m^6^A methyltransferase that epigenetically upregulates ETS proto-oncogene 1 (ETS1) through RNA methylation. This pathway drives oncogenic processes, including cell cycle dysregulation and tumor progression, highlighting WTAP as both a potential therapeutic target and a prognostic indicator in hepatocellular carcinoma [69]. Overall, the diagnostic utility of m^6^A epigenetic regulators stems from their ability to orchestrate epitranscriptomic reprogramming, thereby modulating gene expression landscapes critical for cancer detection. These RNA-modifying enzymes demonstrate exceptional promise as biomarkers across multiple diagnostic platforms, including noninvasive liquid biopsy approaches and tissue-based assays. Dysregulation of m^6^A regulatory machinery enables early cancer detection through aberrant RNA methylation patterns that distinguish malignant cells from their normal counterparts. Liquid biopsy applications leverage cell-free RNA modifications as dynamic biomarkers for tumor burden monitoring and minimal residual disease detection, while tissue profiling provides complementary information for primary diagnosis and metastatic workup.

### Prognostic value of m^6^A modification

The critical prognostic value of m^6^A modification in cancer is increasingly recognized, as dynamic epigenetic alterations in this system serve as independent biomarkers that are predictive of patient survival outcomes and disease recurrence risk across diverse malignancies [248]. Prognostic models incorporating m^6^A enzyme expression profiles, such as the thyroid cancer risk assessment tool and the glioma m^6^AScore system, which link modification patterns to immunotherapy response, have demonstrated predictive accuracy. Clinical correlations revealed that m^6^A alterations are associated with tumor stage, metastatic potential, and survival outcomes across malignancies. Liquid biopsy approaches for detecting circulating tumor RNA methylation enable real-time tumor burden monitoring, whereas tissue-based profiling provides complementary prognostic data. These epigenetic modifications not only serve as independent predictors but also reveal therapeutic vulnerabilities, designating m^6^A as an indispensable prognostic biomarker and a key therapeutic target [249]. Specifically, METTL16 and METTL3 have distinct prognostic implications in pancreatic cancer. METTL16 serves as a favorable prognostic biomarker because of its association with improved survival outcomes, whereas METTL3 may influence tumor progression through RNA metabolic reprogramming [152]. Moreover, upregulated expression of METTL3 in colorectal cancer is associated with an unfavorable prognosis, a more advanced disease stage, and decreased survival rates. Mechanistically, METTL3-mediated m^6^A modification promotes JAK/STAT signaling, driving protumorigenic inflammation and immune evasion [250]. Intriguingly, KIAA1429 stabilizes RAS Guanyl-releasing protein 1 (RASD1) mRNA through m^6^A-dependent regulation, thereby increasing its expression and promoting oncogenic activity. The suppression of RASD1 partially reverses the tumor-promoting impacts of KIAA1429, thereby underscoring its clinical significance as a prognostic biomarker and a prospective therapeutic target in the realm of gastric cancer [73]. Aberrant cytosolic accumulation of KIAA1429 is correlated with aggressive tumor phenotypes and poor patient outcomes in breast cancer. Functional studies have revealed that KIAA1429 depletion significantly impairs cancer cell proliferation, migration, and invasion capacity. Mechanistic investigations demonstrate that cytosolic KIAA1429 interacts with IGF2BP3 to stabilize m^6^A-hypermethylated hyaluronan synthase 2 (HAS2) mRNA, thereby promoting oncogenic hyaluronan synthesis and tumor growth [251]. Notably, FTO modulates immunoediting pathways by upregulating immune checkpoint ligands, fostering an immunosuppressive microenvironment. These dual roles in tumor progression and immune evasion position FTO as both a critical prognostic indicator for improving gastric cancer management [76]. In addition, FTO overexpression in bladder cancer tissues exhibits a significant correlation with aggressive tumor phenotypes and could serve as an independent predictor of clinical outcomes. At the molecular level, FTO-mediated m^6^A demethylation stabilizes oncogenic drivers such as MALAT1 and CDK6, resulting in the formation of regulatory circuits that increase tumor cell proliferation, invasion, and chemoresistance [158]. The FTO-mediated upregulation of platelet-derived growth factor C (PDGFC) autocrine signaling through the m^6^A/YTHDF2 axis drives pancreatic cancer progression. Mechanistically, FTO depletion induces m^6^A hypermethylation at the PDGFC 3'-UTR, promoting YTHDF2-dependent mRNA decay and transcriptional suppression of this oncogenic ligand. The clinical significance of this pathway is highlighted by the potential application of FTO/PDGFC coexpression profiling as a prognostic biomarker and a predictive marker for therapeutic response in pancreatic adenocarcinoma [252]. In addition, ALKBH5 functions as a Pivotal independent prognostic marker in acute myeloid leukemia, with its overexpression associating with unfavorable clinical Outcomes and persistence of leukemia stem cells, thereby implicating its role in therapeutic resistance and disease progression. Its leukemia-specific role in regulating oncogenic targets such as transforming acidic coiled-coil containing protein 3 (TACC3) underscores its therapeutic potential as a targetable vulnerability in acute myeloid leukemia [83]. Overall, the prognostic significance of m^6^A epigenetic dysregulation extends across cancer subtypes, with aberrant expression of m^6^A regulatory enzymes consistently correlating with clinical aggression, therapeutic resistance, and adverse outcomes. These modifications not only serve as independent predictors of survival but also inform treatment stratification by modulating tumor–immune microenvironment interactions and therapeutic vulnerabilities. As actionable biomarkers, m^6^A regulators demonstrate transformative potential in precision oncology, enabling patient risk stratification and guiding targeted intervention strategies.

### m^6^A modification in cancer treatment resistance

The treatment resistance mediated by m^6^A epigenetic dysregulation arises through multilayered molecular orchestration, involving coordinated regulation of drug-metabolizing enzymes, reprogramming of prosurvival signaling cascades, and metabolic adaptation to therapeutic stress. This dynamic interplay across epigenetic, signaling, and metabolic axes collectively drives treatment failure in patients with malignancies. Specifically, METTL3 drives oxaliplatin resistance in hepatocellular carcinoma by activating the G6PD-dependent pentose phosphate pathway. This occurs via METTL3-catalyzed m^6^A modification of the TRIM21 mRNA 3'-UTR, which recruits YTHDF2 to reduce RNA stability [61]. METTL3-induced m^6^A methylation of TP53 reduces chemotherapy sensitivity in hepatocellular carcinoma by destabilizing TP53 mRNA. Inhibition of METTL3 stabilizes TP53, restoring p53-mediated apoptosis and sensitizing cancer cells to chemotherapy [253]. Moreover, the inhibition of METTL3 can increase glioblastoma stem cell self-renewal by modulating m^6^A modification of adenine phosphoribosyltransferase (APNG) mRNAs, thereby increasing the sensitivity of glioblastoma multiforme to temozolomide [254]. The AGD1/USP10/METTL13 complex promotes cancer stem cell proliferation and reduces docetaxel efficacy in castration-resistant prostate cancer by inducing CD44 mRNA m^6^A methylation [255]. In addition, circ0008399 binds to WTAP to promote the formation of the WTAP/METTL3/METTL14 m^6^A methyltransferase complex. circ0008399 increases the expression of TNF alpha-induced protein 3 (TNFAIP3) by increasing its mRNA stability in an m^6^A-dependent manner. Circ0008399 binding to WTAP enhances m^6^A methyltransferase complex assembly and activity, promoting cisplatin resistance in bladder cancer [256]. Additionally, KIAA1429 functions as a novel signaling hub to activate the JNK/MAPK pathway, driving tumorigenesis and gefitinib resistance in lung adenocarcinoma. This axis mediates oncogenic signaling and targeted therapy resistance, highlighting KIAA1429 as a potential therapeutic target [74]. In addition, ALKBH5-mediated m^6^A demethylation of FOXO1 mRNA stabilizes its transcript, restoring redox homeostasis and promoting cancer stem cell traits and doxorubicin resistance in triple-negative breast cancer [257]. ALKBH5 drives glycolysis in resistant breast cancer cells by mediating YTHDF2-dependent m^6^A demethylation of GLUT4 mRNA, which stabilizes the transcript and enhances glucose metabolism [84]. Interestingly, FTO may attenuate gemcitabine resistance in Pancreatic cancer by modulating neural precursor cell expressed developmentally downregulated 4 (NEDD4) mRNA stability through the PTEN/PI3K/AKT signaling axis. In addition, FTO depletion significantly upregulates PTEN expression in a NEDD4-dependent manner, thereby regulating gemcitabine sensitivity via the PI3K/AKT pathway in pancreatic cancer cells [79]. FTO exhibits overexpression in both primary and 5-FU-resistant colorectal cancer. Mechanistically, the pro-apoptotic factor SIVA1 serves as a critical downstream effector of FTO-mediated m^6^A demethylation [258]. Notably, YTHDF1 has been shown to enhance liver cancer stem cell renewal and confer resistance to multiple tyrosine kinase inhibitors including lenvatinib and sorafenib in patient-derived organoid models [216]. Importantly, YTHDF2-mediated m^6^A modification of one cut homeobox 2 (ONECUT2) can promote stemness in gastric cancer. YTHDF2 is downregulated, and its overexpression facilitates ONECUT2 mRNA degradation through m^6^A modification in gastric cancer [259]. Suppression of YTHDC2 in colorectal cancer cells impairs binding to m^6^A modification sites on LiM kinase 1 (LIMK1) mRNA, thereby enhancing its stability and upregulating expression. Aberrant overexpression of LIMK1 drives eIF2α hyperphosphorylation, triggering endoplasmic reticulum stress and enhancing stress granule assembly, which collectively culminates in resistance to 5-FU in colorectal cancer cells [104]. IGF2BP3 functions as an m^6^A reader to stabilize and enhance translation of EGFR mRNA through collaboration with METTL14, thereby activating EGFR signaling and conferring therapeutic resistance to cetuximab in colorectal cancer [260]. In summary, m^6^A-mediated epigenetics in drug resistance predominantly hinges on the aberrant expression of drug-responsive genes and the suppression of diverse stress response signaling cascades. The orchestrated interplay among m^6^A methyltransferases, demethylases, and binding proteins establishes a multifaceted regulatory architecture that finely tunes pharmacokinetic processes and the expression of target pharmacogenes. This regulation ultimately affects cancer cell responses to chemotherapy, radiotherapy, and targeted drugs in a tumor-specific manner.

## Therapeutic targeting of m^6^A modification in cancer

**Fig. 6:**
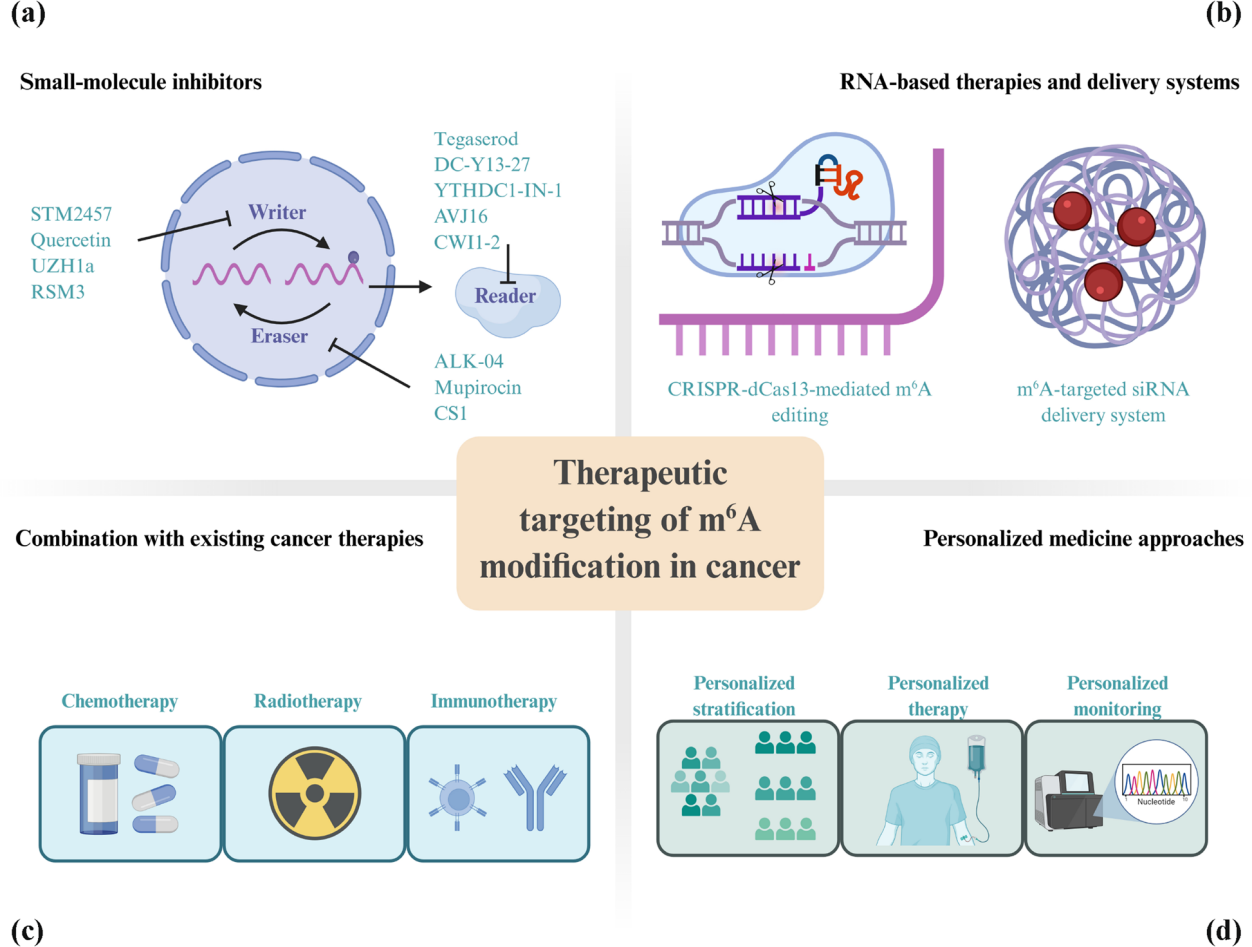
Therapeutic targeting of m^6^A modification in cancer. This figure illustrates the potential of m^6^A modification in cancer therapy, including small-molecule inhibitors, RNA-based therapies and delivery systems, combinations with existing cancer therapies, and personalized medicine approaches. **a** Small-molecule inhibitors include STM2457, Quercetin, UZH1a, and RSM3 for writers, ALK-04, Mupirocin, and CS1 for erasers, Tegaserod, DC-Y13-27, YTHDC1-IN-1, AVJ16, and CWI1-2 for readers. **b** RNA-based therapies and delivery systems include CRISPR-dCas13-mediated m^6^A editing and m^6^A-targeted siRNA delivery system. **c** Combination therapies include m^6^A combination with chemotherapy, radiotherapy and immunotherapy. **d** Personalized medicine includes personalized stratification, personalized therapy and personalized monitoring

m^6^A methylation is emerging as a pivotal therapeutic target in oncology, influencing tumor progression and treatment response. We subsequently analyze innovative strategies including development of m^6^A modification inhibitors, combinations with existing cancer therapies, and personalized medicine approaches to harness this dynamic RNA modification for cancer therapy (Fig. 6).

### Small-molecule inhibitors

Advancement of small-molecule inhibitors directed against m^6^A modifications carries significant translational potential, particularly in oncology and immunology (Table 3). The epitranscriptomic plasticity and reversible epigenetic regulation inherent to m^6^A methylation confer it as a highly attractive target for therapeutic modulation. Specifically, 3-deazaadenosine is a global methylation inhibitor. The application of 3-deazaadenosine decreases flotillin-1 mRNA expression in ovarian cancer cells and suppresses tumor formation in a xenograft mouse model [261]. Moreover, STM2457 has robust efficacy as a selective METTL3 catalytic inhibitor. Compared with the control, STM2457 significantly reduces colorectal cancer tumor growth. Compared with monotherapy, combination treatment with STM2457 and anti-PD1 therapy produces significantly superior antitumor responses characterized by increased infiltration of activated T lymphocytes within tumor allografts [59]. Phase I trials show tolerability and early efficacy signals, with ongoing studies exploring combinations and biomarker-driven strategies. This represents a breakthrough in targeting METTL3-driven malignancies [271]. In addition, quercetin functions as a selective METTL3 inhibitor, exhibiting an IC50 of 2.73 μM, and demonstrates dose-dependent attenuation of m^6^A levels in pancreatic cancer cells [262]. In the context of METTL3-targeted therapeutic interventions, Quercetin has emerged as a multifunctional agent that is currently under investigation across multiple clinical domains, with ongoing trials exploring its utility in Managing anemia associated with type 2 diabetic nephropathy, assessing cognitive enhancement in schizophrenia spectrum disorders through neuroimaging and neuropsychological assessments, and elucidating pharmacokinetic interactions with pravastatin metabolism [272]. Similarly, UZH1a, a well-characterized METTL3 inhibitor, suppresses the invasive and migratory capacities of multiple cancer cell types, including osteosarcoma and acute myeloid leukemia cells, through attenuation of m^6^A methylation [263]. Furthermore, RSM3 serves as a stapled peptide inhibitor for METTL3 and can induce METTL3 degradation and suppress m^6^A mRNA methylation, exhibiting remarkable anticancer efficacy in a prostate cancer cell xenograft tumor model [264]. Additionally, ALK-04 has been characterized as a selective ALKBH5 inhibitor, and ALK-04 treatment significantly reduces melanoma cell growth [265]. Moreover, CS1 is also a specific inhibitor of FTO. CS1 triggers G2/M phase cell cycle arrest and enhances apoptotic processes in colorectal cancer cells [266]. FB23-2 is a first-in-class FTO inhibitor that targets mRNA m^6^A demethylation in acute myeloid leukemia. Phase I trials demonstrated acceptable safety and partial efficacy in subjects with relapsed/refractory acute myeloid leukemia, with phase II clinical trials exploring combinations such as azacitidine/venetoclax [267]. A structure-guided computational screening of FDA-approved pharmacologic agents identified tegaserod as a candidate YTHDF1 inhibitor. This compound prevents direct interaction between YTHDF1 and m^6^A-modified mRNAs, while suppressing YTHDF1-mediated translational regulation of cyclin E2. Furthermore, tegaserod demonstrates attenuated viability in patient-derived acute myelogenous leukemia cells under ex vivo conditions and extends survival duration in patient-derived xenograft models [217]. Interestingly, DC-Y13-27 acts as a specific inhibitor of YTHDF2. Treatment with DC-Y13-27 in aged and H2O2-exposed mice significantly upregulated FOXO3 and TIMP1 expression, attenuated MMP activity, and alleviated intervertebral disc degeneration [268]. YTHDC1-IN-1 functions as a specific inhibitor of YTHDC1 and has antiproliferative effects on acute myeloid leukemia cells [269]. Importantly, AVJ16 is a highly selective and potent IGF2BP1 inhibitor, demonstrates antiproliferative activity in lung cancer cells and triggers apoptotic processes in colorectal cancer cells [270]. CWI1-2 has been identified as a specific IGF2BP2 inhibitor and shown to reduce Gln uptake and impair mitochondrial function, leading to decreased ATP production in acute myeloid leukemia cells [115]. In summary, the development of m^6^A modification inhibitors represents a promising avenue for therapeutic interventions against multiple malignancies. By correcting aberrant RNA processing and restoring cellular homeostasis, these inhibitors hold promise for transforming the landscape of therapeutic paradigms and optimizing clinical efficacy in oncology. However, the translation of these preclinical successes to clinical applicability necessitates rigorous investigations through large-scale, multicenter clinical trials to establish pharmacokinetic profiles, optimize dosing regimens, and evaluate long-term safety outcomes. Current limitations include insufficient characterization of off-target effects, variability in patient response biomarkers, and lack of head-to-head comparisons with established therapies. Future directions should prioritize mechanistic studies elucidating tumor-specific m^6^A signatures, the development of combination regimens with immunotherapy or targeted agents, and the establishment of predictive biomarkers to enable precision medicine approaches in this evolving therapeutic paradigm.

**Table 3:** Inhibitors of m^6^A modification for cancer treatment

Inhibitor	Target	Indications	Clinical trial/development stage	Mechanism faction	Reference
3-deazaadenosine	m^6^A	Ovarian cancer	Preclinical study	It reduces the levels of m^6^A modification	[261]
STM2457	METTL3	Colorectal cancer	Preclinical study	It inhibits METTL3 to reduce m^6^A modification	[59]
Quercetin	METTL3	Pancreatic cancer	Preclinical study	It inhibits METTL3 to reduce m^6^A modification	[262]
UZH1a	METTL3	Osteosarcoma	Preclinical study	It inhibits METTL3 to reduce m^6^A modification	[263]
RSM3	METTL3	Acute myeloid leukemia	Preclinical study	It inhibits METTL3 to reduce m^6^A modification	[264]
ALK-04	ALKBH5	Melanoma	Preclinical study	It inhibits ALKBH5 to increase m^6^A modification	[265]
CS1	FTO	Colorectal cancer	Preclinical study	It inhibits FTO to increase m^6^A modification	[266]
FB23-2	FTO	Acute myeloid leukemia	Clinical trial phase II	It inhibits FTO to increase m^6^A modification	[267]
Tegaserod	YTHDF1	Acute myeloid leukemia	Preclinical study	It inhibits the activity of YTHDF1	[217]
DC-Y13-27	YTHDF2	Disc degeneration	Preclinical study	It inhibits the activity of YTHDF2	[268]
YTHDC1-IN-1	YTHDC1	Acute myeloid leukemia	Preclinical study	It inhibits the activity of YTHDC1	[269]
AVJ16	IGF2BP1	Lung cancer	Preclinical study	It inhibits the activity of IGF2BP1	[270]
CWI1-2	IGF2BP2	Acute myeloid leukemia	Preclinical study	It inhibits the activity of IGF2BP2	[115]

### RNA-based therapies and delivery systems

CRISPR-dCas13-mediated m^6^A editing is an emerging gene editing technology that can precisely regulate m^6^A modification. By combining RNA cleavage-inactive dCas13 with the methyltransferase GhMTA or the demethylase GhALKBH10, a targeted RNA methylation editor (TME) or a targeted RNA demethylation editor (TDE) can be constructed [273]. Interestingly, using the CRISPR/Cas13a system to specifically recognize and bind to RNA, the core functional domain of methyltransferase complex and demethylase can be fused with dCas13 without cleavage activity to develop a new RNA methylation or demethylation editing tool with better editing specificity and fewer off-target effects. CRISPR-dCas13-mediated m^6^A editing technology achieves precise regulation of m^6^A modification of specific RNA by fusing dCas13 with m^6^A modification-related enzymes [274]. Attractively, targeted m^6^A demethylation of centromeric RNA (cenRNA) via the CRISPR-dCas13b-FTO platform can induce centromere structural instability, characterized by impaired kinetochore assembly and chromatin disorganization. This epigenetic perturbation disrupted mitotic progression, leading to apoptotic cell death in malignant cells. Notably, the intervention selectively inhibited oncogenic proliferation while maintaining viability in non-transformed cells, highlighting its potential as a precision RNA epigenetic therapy [275]. Although the application of siRNA drug transport systems in cancer treatment has shortcomings, such as rapid degradation and limited targeting specificity, recent research has combined nanotechnology with drug delivery systems to achieve the delivery of lipid nanoparticles (LNPs) and polymer nanoparticles (NPs) carrying siRNA [276, 277]. Importantly, LNPs demonstrate efficient delivery of circSCMH1 RNA to the brain via intranasal administration, enabling targeted modulation of FTO-dependent m^6^A epitranscriptomic remodeling. This system specifically reduces m^6^A modification on phospholipid phosphatase 3 (PLPP3) mRNA establishing a mechanistic foundation for developing siRNA-based therapeutic strategies against m^6^A-related neurological disorders. Notably, the versatility of nanocarrier platforms extends beyond nucleic acid delivery, as evidenced by their capacity to encapsulate diverse therapeutics including small-molecule inhibitors and gene-editing tools [278]. Moreover, pH-sensitive nanoliposomes engineered with tumor-microenvironment-responsive ligands demonstrate selective internalization by MDSCs via receptor-mediated endocytosis. This targeted delivery system suppresses MDSC immunosuppressive activity through metabolic reprogramming, thereby restoring cytotoxic T-cell functionality. Consequently, the system significantly inhibits hepatocellular carcinoma stem cell proliferation, migration, and invasive potential in preclinical models [279]. Besides, the shell of nanoparticles can reduce the degradation rate of the bound siRNA and release it at the tumor lesion after targeted transport, thereby increasing the drug concentration in the lesion area. METTL3-catalyzed m^6^A modification upregulates the adipogenesis-related lncRNA LINCO0958, establishing its therapeutic potential for addressing liver cancer dysregulation through RNA epigenetic modulation. siLINC00958-loaded nanoparticles demonstrated precise tumor targeting, controlled siRNA release, and dose-dependent inhibition of hepatocellular carcinoma cell proliferation, highlighting the translational potential of this integrated delivery-epigenetic intervention strategy [280]. Taken together, with the iterative optimization of technological platforms, the CRISPR-dCas13-mediated m^6^A epigenetic editing system integrated with m^6^A-targeted siRNA delivery modules will assume a progressively pivotal role in combinatorial immunotherapy, personalized treatment regimens, and precision oncology paradigms, thereby offering novel therapeutic prospects for cancer management.

### Combinations with existing cancer therapies

Combining treatments targeting this modification with existing cancer therapies, such as chemotherapy, radiotherapy, and immunotherapy, can improve treatment efficacy by modulating immune responses and metabolic pathways. This approach also has the potential to address drug resistance and improve overall survival integrated with chemotherapy, the synergistic integration of m^6^A modification and the anticancer drug 5-azacytidine can effectively target cancer stem cells, impairing their capacity to drive tumor progression dynamics and metastatic dissemination, with notable relevance in highly aggressive molecular subtypes of breast cancer [281]. Moreover, BRD2 m^6^A modification can enhances the chemosensitivity of gastric cancers to 5-FU via the caspase-8 activation and the induction of apoptosis and pyroptosis [282]. In addition, targeting the FTO and PDK1/AKT signaling axes with FB23 and BX-912 suppresses breast carcinoma progression, augments cytotoxic T-lymphocyte functionality, and synergistically enhances the therapeutic efficacy of the anti-PD-1/PD-L1 immunotherapy atezolizumab in preclinical models [283]. In combination with radiotherapy, the downregulation of FTO expression or treatment with the FTO inhibitor FB23-2 combined with radiotherapy significantly inhibits glioblastoma stem cell proliferation and self-renewal and increases apoptosis. FB23-2 in combination with radiotherapy demonstrates synergistic inhibition of intracranial tumor growth and significantly prolongs survival in tumor-bearing murine models [267]. Furthermore, IGF2BP2 activates the m^6^A–SLC1A5–mTORC1 signaling axis, thereby enhancing glutamine uptake and promoting pancreatic cancer progression. Silencing IGF2BP2 enhances radiosensitivity in pancreatic cancer [116]. In addition, METTL3 activity dynamically responds to ionizing radiation, potentially mediating apoptotic cell death triggered by radiation through epigenetic regulation. This process reverses miR-20b-induced suppression of BNIP2 via YTHDC1-dependent m^6^A modification of the lncRNA MEG3 [284]. In combination with immunotherapy, the high matrix stiffness in pancreatic ductal adenocarcinoma stabilizes IGF2BP2, which subsequently promotes sphingomyelin synthesis via SGMS2 upregulation. This pathway can facilitate PD-L1 localization on membrane lipid rafts, enhancing immune evasion. The disruption of sphingomyelin synthesis improves antitumor immunity and suppresses tumor growth in humanized mice, highlighting immunotherapeutic opportunities for pancreatic ductal adenocarcinoma [285]. Interestingly, ALKBH5 exhibits a tumor-suppressive role as a biomarker and correlates with immunotherapy responsiveness in hepatocellular carcinoma. Mechanistically, ALKBH5 modulates the tumor immune microenvironment and immunotherapy responsiveness through targeting TIM3. This study provides novel insights into the interplay between m^6^A modification and immune checkpoint inhibitors, offering potential therapeutic implications for hepatocellular carcinoma patients [286]. Crucially, YTHDF2 has the ability to facilitate immune escape through destabilizing mRNAs. Targeting YTHDF2 with small molecules can curb aggressive B-cell malignancies and enhance their susceptibility to CAR-T cell therapy [287]. In terms of overcoming drug resistance, the interaction of phosphoglycerate dehydrogenase (PHGDH) with IGF2BP1 can facilitate the m^6^A-dependent stabilization of transcription factor 7 Like 2 (TCF7L2) mRNA to confer multidrug resistance in gastric cancer [288]. Notably, the methylation of METTL3, which is mediated by PRMT5, might foster cisplatin resistance in ovarian cancer by bolstering DNA repair pathways [289]. Moreover, CREB regulated transcription coactivator 2 (CRTC2) can form condensates with YTHDF2, thereby augmenting the translational efficiency of m^6^A-modified mRNAs and subsequently promoting hepatocarcinogenesis alongside lenvatinib resistance [290]. In conclusion, the integration of m^6^A-targeted strategies with existing cancer therapies may represent a transformative paradigm in cancer treatment. By modulating RNA metabolism, m^6^A regulators can increase therapeutic sensitivity, overcome resistance mechanisms, and synergize with immunotherapy by increasing antigen presentation.

### Personalized medicine approaches

The advent of personalized medicine has revolutionized cancer treatment by tailoring therapies to individual patient characteristics, tumor biology, and molecular profiles. Among the emerging biomarkers, m^6^A modification stands out as a dynamic epigenetic regulator with profound implications for precision oncology [291]. Tumor-specific m^6^A patterns may create heterogeneous RNA landscapes that determine drug sensitivity, immune responsiveness, and metastatic potential [292]. First, patient stratification via m^6^A profiling may represent a transformative approach in oncology, utilizing cutting-edge next-generation sequencing technologies to quantitatively map m^6^A epitranscriptomes from clinical biopsy samples and thereby identify distinct molecular subtypes with unique therapeutic vulnerabilities that inform personalized treatment strategies. For example, patients with breast cancer whose tumors display hypermethylated m^6^A patterns within immunoregulatory genes may experience improved treatment outcomes when immunotherapies combining immune checkpoint inhibitors with targeted m^6^A inhibitors are administered [293]. Second, cancer patients can receive personalized combination therapy based on the specificity of their individual m^6^A methylation profile. The dynamic field of epitranscriptomics has experienced remarkable progress throughout the evolution of m^6^A-targeted therapeutic interventions, specifically via the design and rigorous clinical assessment of small—molecule inhibitors and activators that target m^6^A—regulatory enzymes. These agents constitute a novel class of therapeutics that aim to correct aberrant m^6^A patterns, thereby restoring normal cellular function and disrupting oncogenic processes. For example, targeting the FTO demethylase in leukemia patients with specific m^6^A patterns may restore tumor suppressor expression. These modulators show promise for correcting aberrant m^6^A patterns, disrupting oncogenic processes, and integrating with existing therapies such as immunotherapy to enhance efficacy, highlighting the future of precision oncology [294]. Third, the response of cancer patients to treatment can be dynamically monitored through changes in m^6^A modification profiles. Innovative liquid biopsy approaches are revolutionizing the real-time assessment of therapeutic efficacy and the emergence of resistance mechanisms in cancer patients. By analyzing circulating tumor RNA for m^6^A alterations, clinicians can gain critical insights into treatment-induced molecular changes. This noninvasive method allows longitudinal tracking of m^6^A patterns, which are increasingly recognized as biomarkers of treatment response and disease progression. For example, shifts in the m^6^A methylation status at specific gene loci may correlate with drug sensitivity or resistance, enabling timely adjustments to therapeutic regimens. The integration of m^6^A-focused liquid biopsies into clinical decision-making frameworks holds significant promise for optimizing treatment outcomes and advancing personalized oncology [288]. Overall, the m^6^A epitranscriptome offers a promising field for the advancement of next-generation personalized cancer treatment approaches. Through unraveling the intricate interaction between m^6^A modification and tumor biology, we can transform cancer treatment approaches to individualized care. Future efforts should focus on translating m^6^A discoveries into actionable diagnostics and therapeutics while addressing the challenges related to biomarker standardization and combinatorial treatment optimization.

## Conclusions and perspectives

m^6^A modification has emerged as a pivotal factor, presenting fresh viewpoints and viable therapeutic targets within the context of cancer treatment. m^6^A methylation levels not only correlate with tumor histopathological classification and prognostic outcomes but also exert regulatory functions across distinct phases of tumorigenesis, progression, and metastatic dissemination. Specifically, m^6^A modification can promote carcinogenesis by regulating oncogenes, influencing cancer stem cells, and modulating the TME. Moreover, m^6^A methylation can affect angiogenesis and immune responses, and tumor growth. In the clinical field, small-molecule inhibitors targeting m^6^A are being tested for efficacy and safety. The combined application of m^6^A-targeted therapy and existing cancer therapies also has promising prospects. Therapeutic strategies based on m^6^A modification are expected to achieve enhanced efficacy with fewer side effects by precisely targeting tumor-specific pathways, influencing immune regulation, and enabling the implementation of personalized interventions. In the future, further integration of multiomic data is needed to accelerate clinical translation and achieve true precision medicine. Given that m^6^A modification plays important roles in cancerous tissues as well as in normal tissues, m^6^A modification strategies for cancer treatment with dual benefits have significant potential. Targeted inhibition of m^6^A regulatory factors implicated in oncogenic transformation represents a highly promising therapeutic paradigm, as it enables selective attenuation of neoplastic proliferation while preserving the physiological homeostasis of non-malignant tissues.

The precise role of m^6^A regulatory proteins in modulating responses across distinct tumor types and stages represents a critical area of investigation in cancer. However, the temporal dynamics of their regulatory actions, the specific m^6^A modifications they mediate within disease-relevant transcripts, and the upstream molecular determinants governing their expression and activity in heterogeneous tumor contexts remain incompletely characterized. Numerous factors regulate m^6^A modification, and precisely targeting these factors for functional precision therapy is a key focus of future research. Additionally, the clinical implementation of m^6^A methylation remains nascent, with therapeutic agents targeting its regulatory machinery currently confined to preclinical investigation. Translating fundamental research discoveries into clinical practice constitutes a formidable challenge. More clinical trials are needed to evaluate the safety and efficacy of m^6^A-related targeted therapies. Therefore, future research should focus on developing more precise m^6^A detection technologies to achieve the accurate quantification and localization of m^6^A modification sites. In response to the heterogeneity of cancer, a pancancer analysis should be conducted to investigate the cell-specific regulatory networks of m^6^A in different cancer types and cell types in detail. Clarifying the differences and commonalities of m^6^A modifications between different tumor tissues and their relationships with tumor heterogeneity will provide a basis for personalized treatment.

In conclusion, m^6^A modification merits further investigation as a pivotal determinant of tumor pathogenesis and therapeutic responsiveness. Despite the multifaceted challenges inherent in m^6^A research, the integration of diverse therapeutic modalities, coupled with the promotion of interdisciplinary collaboration and technological innovation, holds promise in driving transformative advancements in cancer treatment. Such progress may yield more favorable prognostic outcomes, substantially improve treatment efficacy, increase patient quality of life, and unlock novel avenues and expansive horizons in oncology.

## Data Availability

Not applicable.
